# Comprehensive Analysis of the Association Between the rs1138272 Polymorphism of the GSTP1 Gene and Cancer Susceptibility

**DOI:** 10.3389/fphys.2018.01897

**Published:** 2019-01-25

**Authors:** Fei Ding, Jin-Ping Li, Yong Zhang, Guang-Hui Qi, Zhi-Chao Song, Yong-Hua Yu

**Affiliations:** ^1^Department of Radiation Oncology, Shandong Cancer Hospital Affiliated to Shandong University, Shandong University, Jinan, China; ^2^Second Department of Oncology, The First Hospital of Zibo, Zibo, China; ^3^Department of Public Health, The First Hospital of Zibo, Zibo, China; ^4^Shandong Academy of Medical Sciences, Jinan, China; ^5^Department of Urology Surgery, The First Hospital of Zibo, Zibo, China; ^6^Department of Anorectal Surgery, The First Hospital of Zibo, Zibo, China

**Keywords:** *GSTP1*, polymorphism, cancer, meta-analysis, risk

## Abstract

**Background:** We obtained conflicting results regarding the relationship between the genetic role of the rs1138272 C/T polymorphism of the *GSTP1* (Glutathione *S*-Transferase pi) gene and the risk of various cancers.

**Methods:** Using the presently available data, a meta-analysis was conducted to comprehensively evaluate the genetic relationship between the *GSTP1* rs1138272 polymorphism and cancer susceptibility.

**Results:** A total of 43 studies including 15,688 cases and 17,143 controls were recruited into our quantitative synthesis. In the overall population, we observed an increased risk of overall cancer cases, compared with unrelated controls, in the genetic models of allele T vs. allele C (*P*-association = 0.007, OR = 1.17), carrier T vs. carrier C (*P*-association = 0.035, OR = 1.11), TT vs. CC (*P*-association = 0.002, OR = 1.45), TT vs. CC+CT (*P*-association = 0.009, OR = 1.42), and CT+TT vs. CC (*P*-association = 0.027, OR = 1.13). We detected similar positive results within the Asian population. Additionally, there was a significant increase in the incidence of cancer for Africans under all genetic models (all *P*-association < 0.05, OR > 1). When targeting the Caucasian population, we detected a positive association with the TT vs. CC and TT vs. CC+CT models in the “Colorectal cancer” (*P*-association < 0.05, OR < 1) and “Head and neck cancer” (*P*-association < 0.05, OR > 1) subgroups. For the “Lung cancer” subgroup, we observed a slightly increased risk in Caucasians under the models of allele T vs. allele C, carrier T vs. carrier C, CT vs. CC, and CT+TT vs. CC (*P*-association < 0.05, OR > 1).

**Conclusion:** The TT genotype of the *GSTP1* rs1138272 polymorphism is likely related to the susceptibility to overall cancer in the Asian and African populations and, specifically, “Colorectal” and “Head and neck” cancers in the Caucasian population. In addition, the CT genotype of the *GSTP1* rs1138272 polymorphism may be linked to the risk of lung cancer in Caucasians. Additional evidence is required to confirm this conclusion.

## Introduction

The human *GSTP1* gene is located on chromosome 11 (11q13.2) (Sharma et al., 2017), and the GSTP1 (Glutathione *S*-Transferase pi) protein participates in the drug resistance process of cancer cells (Singh, 2015). Two commonly occurring polymorphisms within the exon 5/6 region of the *GSTP1* gene, namely, rs1695 (A313G, IIe105Val) and rs1138272 (C341T, Ala114Val), may be related to the occurrence and development of certain diseases (Huang et al., 2013; Xie et al., 2014; Tan and Chen, 2015; Zhou et al., 2015; Wang et al., 2016). For instance, the *GSTP1* rs1695 polymorphism is likely associated with the risk of Alzheimer’s disease, based on a previous meta-analysis (Wang et al., 2016). There have been several comprehensive analyses concerning the potential role of the *GSTP1* rs1695 polymorphism in the susceptibility to cancer. However, the results varied between cancer types. For example, *GSTP1* rs1695 was reported to be associated with the risk of esophageal cancer and malignant melanoma in the Caucasian population (Tan and Chen, 2015; Zhou et al., 2015), but not childhood acute lymphoblastic leukemia (ALL) (Zhao et al., 2018) or bladder cancer (Yu et al., 2016). To the best of our knowledge, very limited comprehensive analyses on the relationship between *GSTP1* rs1138272 and overall cancer risk have been reported.

Huang et al. (2013), one relevant meta-analysis containing 28 case-control studies was reported, assessing the potential effect of the *GSTP1* rs1138272 C/T polymorphism on the risk of overall cancer. In view of the publication of new relevant articles in the last 5 years, we performed an updated meta-analysis to gain insight into the genetic association between the rs1138272 C/T polymorphism of the *GSTP1* gene and the risk of cancer. Altogether, 43 eligible case-control studies were recruited into our statistical analysis.

## Materials and Methods

### Database Searching

Five online databases extending until September 2018, including PubMed, Embase, Cochrane, Scopus, and WOS (web of science), were utilized for the article identification. Referring Preferred Reporting Items for Systematic Reviews and Meta-Analyses (PRISMA) (Moher et al., 2009) were considered. The search terms are shown in Supplementary Table S1.

### Screening Process

First, duplicate articles or articles with overlapping data were removed. In addition, review articles, meta-analyses, meeting abstracts and case reports were excluded. Articles that lacked normal control data or the complete genotype data on the CC, CT, TT status of *GSTP1* rs1138272 in the cases/controls were also removed. The basic information was then collected and summarized, and *P*-HWE (*P*-value for Hardy–Weinberg equilibrium) was calculated. The quality appraisal of each study was also performed using the Newcastle-Ottawa Scale (NOS) system. Articles with *P*-HWE > 0.05 for the controls and an NOS score > = 5 were included. Eligible case-control studies were finally considered.

### Statistical Tests

A fixed-effects model was applied in the Mantel–Haenszel statistics of association test when the *P*-heterogeneity of Cochran’s Q statistic was larger than 0.1 or the I^2^ value was less than 50%. When those criteria were not met, a random effects model was used in the DerSimonian and Laird statistics of association test. For the assessment of the pooled effect size, we obtained the odds ratio (OR), 95% confidence interval (CI) and *P*-Association (*P*-value of association test) from each meta-analysis and subsequent subgroup analysis by ethnicity, control source, or cancer type.

We used the Begg’s and Egger’s tests to assess the potential publication bias when the number of enrolled case-control studies was larger than 10. We also performed a sensitivity analysis to evaluate the data stability and possible sources of heterogeneity. The STATA software (version 12.0, StataCorp, United States) was used to analyze the following genetic models in the association test, Begg’s test, Egger’s test, and sensitivity analysis: the allele model (allele T vs. allele C), homozygote model (TT vs. CC), heterozygote model (CT vs. CC), dominant model (CT+TT vs. CC), recessive model (TT vs. CC+CT), and carrier model (carrier T vs. carrier C).

## Results

### Case-Control Study Recruitment

**FIGURE 1:**
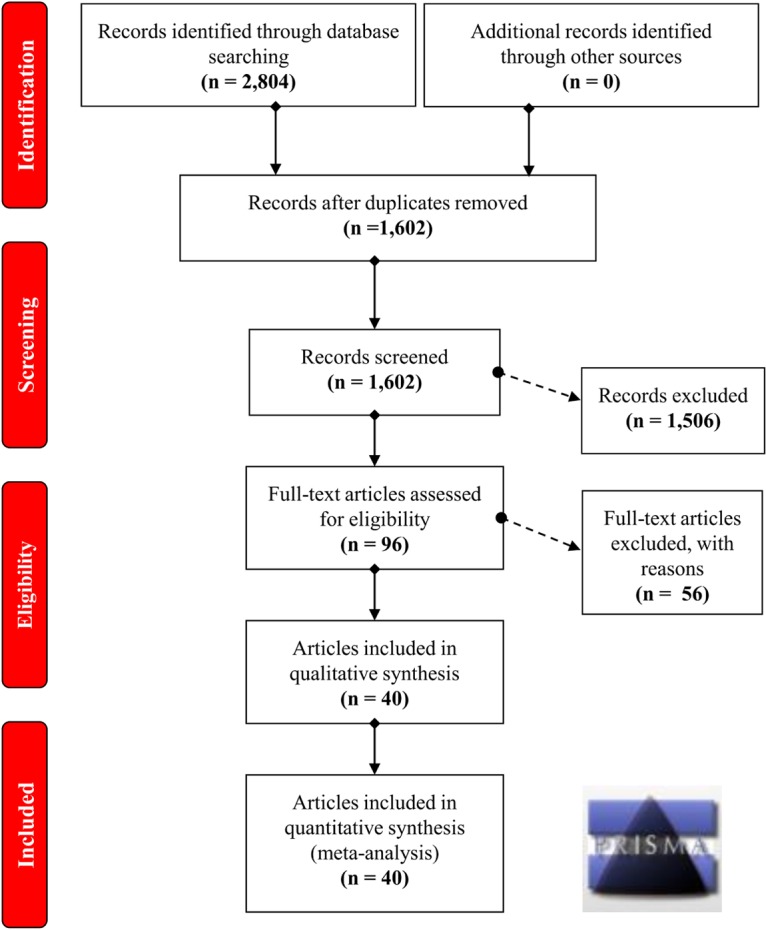
Selection process for eligible case-control studies.

A flow chart illustrating the process of study selection is presented in Figure 1. Briefly, we initially obtained a total of 2,804 records by searching five databases, including 736 records from PubMed, 484 records from Embase, 60 records from Cochrane, 723 records from Scopus and 801 records from WOS. Then, we removed 1,202 duplicate records and excluded the following 1,506 records: 158 reviews; 70 meta-analyses; 42 case reports; 160 meeting abstracts; 62 articles with data on mouse, rat or dog models; 111 articles with *in vitro* data on cell lines; 792 articles focusing on other diseases, other genes or other variants of the *GSTP1* gene; and 111 articles containing data on methylation or gene expression. Next, we assessed the eligibility of the remaining 96 full-text articles. An additional 56 articles were excluded, including 56 articles with unavailable data on the genotype frequency of CC, CT, and TT within *GSTP1* rs1138272, and 4 articles in which the data were not in HWE. After a quality evaluation, 40 articles (Harris et al., 1998; Saarikoski et al., 1998; Park et al., 1999; Wadelius et al., 1999; Welfare et al., 1999; Marshall et al., 2000; Stanulla et al., 2000; Krajinovic et al., 2002; Wang et al., 2003, 2011; Sorensen et al., 2004; Yang et al., 2004; Garcia-Closas et al., 2005; Landi et al., 2005, 2007; Moore et al., 2005; De Roos et al., 2006; Lira et al., 2006; Marciniak et al., 2006; Jiao et al., 2007; Li et al., 2007, 2010; Murphy et al., 2007; Al-Dayel et al., 2008; Kury et al., 2008; Siraj et al., 2008; Van Emburgh et al., 2008; Zienolddiny et al., 2008; Canova et al., 2009; Northwood et al., 2010; Sainz et al., 2011; Ebrahimkhani et al., 2012; Garcia-Gonzalez et al., 2012; Ibarrola-Villava et al., 2012; Dura et al., 2013; Oskina et al., 2014; Ghatak et al., 2016; De Mattia et al., 2017; Minina et al., 2017; Rajesh et al., 2018) of good quality were included. Finally, we included a total of 43 case-control studies for our quantitative synthesis. All of the data in these articles were in HWE. The detailed characteristics of these articles are provided in Table 1.

**Table 1:** Characteristics of the included studies.

First author	Year	Ethnicity	CC-CT-TT (case)	Cancer type	CC-CT-TT (control)	*P*-HWE	Source	Quality	Genotyping
Al-Dayel	2008	Asian	113-24-8	Lymphoma	389-113-8	0.95	PB	6	PCR-RFLP
Canova	2009	Caucasian	1298-193-10	UADT	1249-189-11	0.20	PB/HB	6	APEX
De Mattia	2017	Caucasian	172-13-1	Liver cancer	182-10-0	0.71	PB	7	Pyrosequencing
De Roos	2006	Caucasian	621-109-4	Lymphoma	537-83-6	0.17	PB	7	PCR
Dura	2013	Caucasian	354-66-3	Esophageal cancer	485-91-5	0.46	PB	7	PCR
Ebrahimkhani	2012	Asian	53-19-1	Colorectal cancer	83-12-0	0.51	HB	6	Pyrosequencing
Garcia	2005	Caucasian	966-113-4	Bladder cancer	917-85-5	0.05	HB	6	Mixed assays
Garcia	2012	Caucasian	500-56-1	Gastric cancer	500-57-0	0.20	PB	9	PCR-RFLP
Ghatak	2016	Asian	44-20-16	Gastric cancer	68-12-0	0.47	PB	7	PCR-RFLP
Harris	1998	Caucasian	113-17-1	Colorectal cancer	170-29-0	0.27	PB	7	PCR-RFLP
			154-28-2	Lung cancer	170-29-0	0.27	PB	7	PCR-RFLP
Ibarrola	2012	Caucasian	516-38-1	Skin cancer	314-18-0	0.61	HB	5	TaqMan
Jiao	2007	Caucasian	286-46-3	Pancreatic cancer	242-55-1	0.25	PB	7	Masscode system
Krajinovic	2002	Caucasian	254-24-0	Leukemia	264-36-2	0.53	PB	6	ASO hybridization
Kury	2008	Caucasian	882-137-4	Colorectal cancer	966-146-9	0.19	PB	7	Fluorescent multiplex PCR
Landi	2005	Caucasian	325-35-0	Colorectal cancer	291-32-2	0.29	HB	5	APEX
Landi	2007	Caucasian	80-7-1	MPM	353-36-2	0.31	PB/HB	7	APEX
Li	2010	African	85-49-7	Esophageal cancer	163-21-2	0.17	HB	6	PCR-RFLP
Li	2007	Caucasian	678-114-11	Head and neck cancer	723-109-6	0.40	PB	8	PCR-RFLP
Lira	2006	Caucasian	99-8-0	Skin cancer	112-18-0	0.40	HB	6	PCR-SSCP
Marciniak	2006	Caucasian	81-15-7	Thyroid cancer	42-10-1	0.66	PB	7	PCR-RFLP
Marshall	2000	Caucasian	35-13-0	Skin cancer	155-19-0	0.45	HB	6	PCR-SSCP
Minina	2017	Caucasian	286-62-5	Lung cancer	239-56-5	0.42	PB	7	PCR
Moore	2005	Mixed	591-103-6	Colorectal cancer	596-114-4	0.56	PB	7	TaqMan
Murphy	2007	Caucasian	170-34-3	Esophageal cancer	190-31-2	0.56	PB	8	Multiplex PCR
Northwood	2010	Caucasian	254-53-1	Colorectal cancer	233-60-3	0.69	PB	8	Multiplex PCR
Oskina	2014	Caucasian	305-66-3	Prostate cancer	277-60-6	0.20	PB	6	TaqMan
Park	1999	African	47-3-1	Oral cancer	81-2-0	0.91	HB	6	PCR-RFLP
		Caucasian	93-8-2	Oral cancer	139-23-1	0.96	HB	6	PCR-RFLP
Rajesh	2018	Asian	67-18-5	Oral cancer	167-12-1	0.15	PB	9	PCR-RFLP
Saarikoski	1998	Caucasian	169-36-1	Lung cancer	241-51-1	0.35	PB	6	PCR-RFLP
Sainz	2011	Caucasian	1480-275-10	Colorectal cancer	1472-291-21	0.13	PB	7	KASPar assay
Siraj	2008	Asian	30-8-2	Thyroid cancer	389-113-8	0.95	PB	6	PCR-RFLP
Sorensen	2004	Caucasian	216-36-1	Lung cancer	224-38-4	0.12	PB	6	PCR
Stanulla	2000	Caucasian	52-11-1	Leukemia	48-16-0	0.25	HB	6	PCR-RFLP
Van	2008	Caucasian	328-56-2	Breast cancer	337-47-1	0.63	HB	6	PCR-SSCP
		African	49-5-0	Breast cancer	70-4-0	0.81	HB	6	PCR-SSCP
Wadelius	1999	Caucasian	143-25-3	Prostate cancer	120-28-0	0.20	PB	5	PCR-SSCP
Wang	2011	Asian	261-38-3	Colorectal cancer	263-27-1	0.73	PB	7	PCR-RFLP
Wang	2003	Caucasian	468-108-3	Lung cancer	511-84-3	0.82	PB	7	PCR-RFLP
Welfare	1999	Caucasian	167-28-1	Colorectal cancer	148-25-0	0.31	PB	6	PCR-RFLP
Yang	2004	Mixed	192-32-5	Lung cancer	189-38-2	0.55	PB	6	PCR
Zienolddiny	2008	Caucasian	250-60-9	Lung cancer	333-46-2	0.76	PB	7	APEX


*UADT, upper aerodigestive tract cancer; MPM, malignant pleural mesothelioma; HWE, Hardy–Weinberg equilibrium; PB, population-based control; HB, hospital-based control; PCR, polymerase chain reaction; RFLP, restriction fragment-length polymorphism; SSCP, single-strand conformation polymorphism; APEX, arrayed primer extension technique; ASO, allele-specific oligonucleotide.*

### Meta-Analysis Results

First, we analyzed the relationship between the *GSTP1* rs1138272 polymorphism and the risk of cancer through a meta-analysis of the overall population. As shown in Table 2, a total of 43 case-control studies with 15,688 cases and 17,143 controls were enrolled for the models of allele T vs. allele C, carrier T vs. carrier C, CT vs. CC, CT+TT vs. CC; in addition, 40 studies with 15,479 cases and 16,765 controls were enrolled for the models of TT vs. CC and TT vs. CC+CT. Because there was not a high degree of heterogeneity observed in the homozygote and recessive genetic models, a fixed-effects model was used in the Mantel–Haenszel statistics of association test for those genetic models. For the other genetic models, a random effects model was used in the DerSimonian and Laird statistics of association test. The quantitative synthesis results (Table 2) revealed an increased risk for cancer, compared with the control group, for the genetic models of allele T vs. allele C (*P*-association = 0.007, OR = 1.17), carrier T vs. carrier C (*P*-association = 0.035, OR = 1.11), TT vs. CC (*P*-association = 0.002, OR = 1.45), TT vs. CC+CT (*P*-association = 0.009, OR = 1.42), and CT+TT vs. CC (*P*-association = 0.027, OR = 1.13). Nevertheless, no significant effect on cancer risk was observed for the model of CT vs. CC (Table 2, *P*-association = 0.106). Supplementary Figures S1–S4 presents the forest plot data under the allele, carrier, heterozygote and dominant models. In summary, the TT genotype of the *GSTP1* rs1138272 polymorphism may be associated with an increased susceptibility to cancer.

**Table 2:** Meta-analysis of the overall population.

Models	Study(N)	Case(N)	Control(N)	I^2^	*P*-heterogeneity	Fixed/random	OR [95% CI]	*P*-association
Allele T vs. allele C	43	15,688	17,143	67.1%	<0.001	Random	1.17 [1.04–1.31]	0.007
Carrier T vs. carrier C	43	15,688	17,143	47.9%	<0.001	Random	1.11 [1.02–1.22]	0.035
TT vs. CC	40	15,479	16,765	28.6%	0.049	Fixed	1.45 [1.14–1.83]	0.002
TT vs. CC+CT	40	15,479	16,765	25.0%	0.080	Fixed	1.42 [1.12–1.80]	0.009
CT vs. CC	43	15,688	17,143	52.5%	<0.001	Random	1.09 [0.98–1.21]	0.106
CT+TT vs. CC	43	15,598	16,963	61.4%	<0.001	Random	1.13 [1.01–1.27]	0.027


*N, number; *P*-heterogeneity, *P*-value of heterogeneity test; OR, odds ratio; CI, confidence interval; *P*-association, *P*-value of association test.*

### Subgroup Analysis Results

**FIGURE 2:**
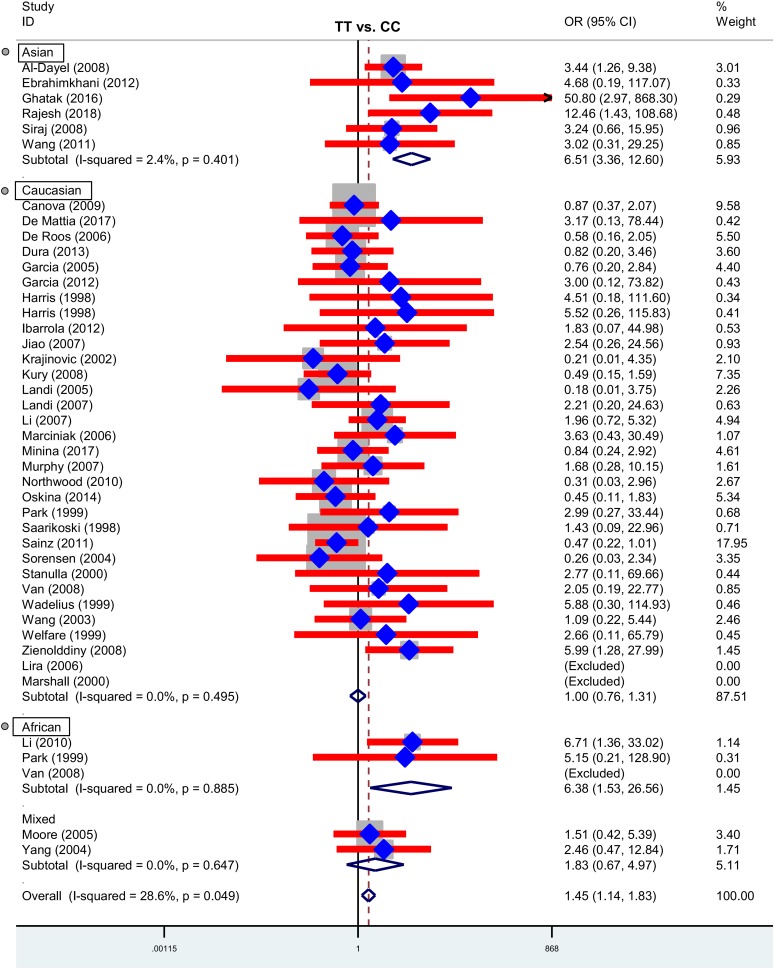
Forest plot of the subgroup analysis by ethnicity in the overall population (TT vs. CC model).

**FIGURE 3:**
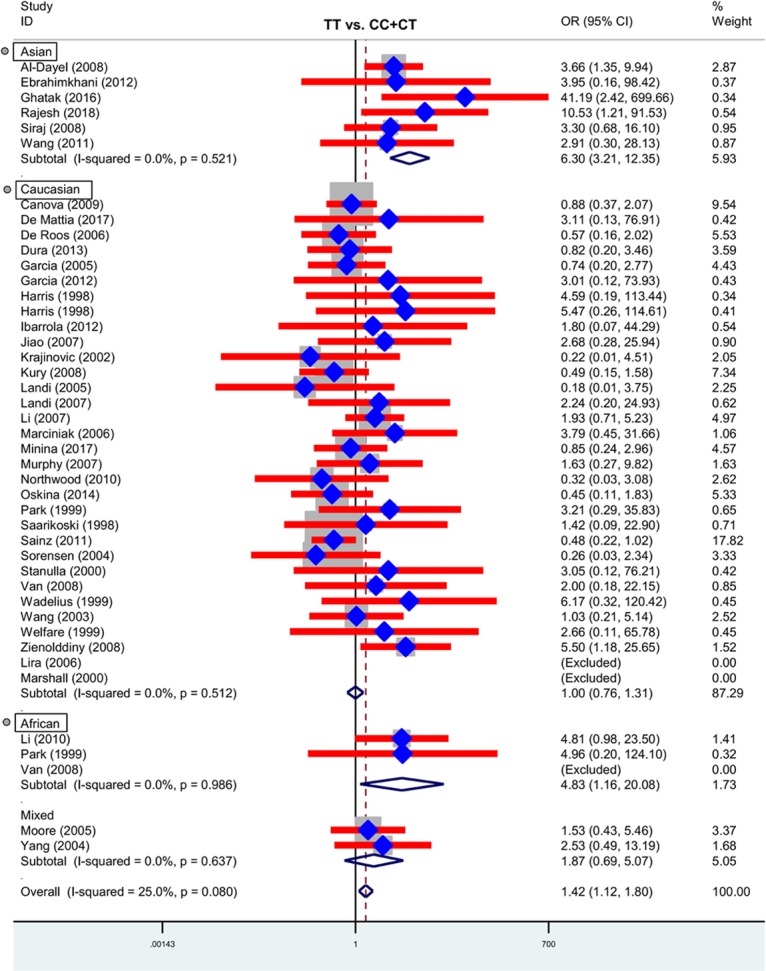
Forest plot of the subgroup analysis by ethnicity in the overall population (TT vs. CC+CT model).

Next, we performed three subgroup analyses based upon ethnicity (Table 3), control source (Supplementary Table S2) and cancer type (Supplementary Table S3) in the overall population. As shown in Table 3, similar positive results were detected in the subgroup “Asian” under the allele, homozygote, recessive and dominant models (Table 3, all *P*-association < 0.05, OR > 1). As shown in Supplementary Table S2, we also assessed the difference between cancer cases and PB-based controls under the TT vs. CC (*P*-association = 0.006, OR = 1.45) and TT vs. CC+CT (*P*-association = 0.007, OR = 1.44) models. Figures 2, 3 present the relative forest plot of the subgroup analysis by ethnicity under the TT vs. CC and TT vs. CC+CT models, while Supplementary Figure S5 shows the forest plot of subgroup analysis by control source under the TT vs. CC model. Moreover, compared with the controls, an increased cancer risk was observed in the “African” subgroup under all of the genetic models (Table 3, all *P*-association < 0.05, OR > 1), but this was not the case for the “Caucasian” (Table 3) and “Hospital-based, HB” (Supplementary Table S2) subgroups (all *P*-association > or = 0.05). In addition, no significant association was found based on cancer type under most of the genetic models, except for the TT vs. CC (Supplementary Table S3, *P*-association = 0.001, OR = 3.11) and TT vs. CC+CT (*P*-association = 0.001, OR = 3.07) models of the “Head and neck cancer” subgroup. Supplementary Figure S6 presents the forest plot of subgroup analysis by cancer type in the overall population under the allele T vs. allele C model, and Supplementary Table S3 shows the pooled data of the “Colorectal cancer” subgroup with nine case-control studies (4,858 cases and 4,998 controls), the “Lung cancer” subgroup with seven case-control studies (2,123 cases and 2,266 controls) and the “Head and neck cancer” subgroup with six case-control studies (1,190 cases and 1,827 controls). Therefore, the rs1138272 polymorphism of the *GSTP1* gene appears to be correlated with an increased risk of cancer in the Asian and African populations. Moreover, the TT genotype of *GSTP1* rs1138272 may be associated with the risk of head and neck cancer in the overall population.

**Table 3:** Subgroup analysis by ethnicity in the overall population.

Subgroup	Models	Study(N)	Case(N)	Control(N)	I^2^	*P*-heterogeneity	OR [95% CI]	*P*-association
Asian	Allele T vs. allele C	6	730	1,666	82.4%	<0.001	2.20 [1.26–3.84]	0.006
	Carrier T vs. carrier C	6	730	1,666	71.4%	0.004	1.81 [1.12–2.93]	0.015
	TT vs. CC	6	730	1,666	2.4%	0.401	6.51 [3.36–12.60]	<0.001
	TT vs. CC+CT	6	730	1,666	0.0%	0.521	6.30 [3.21–12.35]	<0.001
	CT vs. CC	6	730	1,666	72.3%	0.003	1.61 [0.96–2.73]	0.074
	CT+TT vs. CC	6	730	1,666	78.9%	<0.001	1.98 [1.13–3.50]	0.018
Caucasian	Allele T vs. allele C	32	1,3783	1,4191	30.9%	0.051	1.04 [0.95–1.13]	0.406
	Carrier T vs. carrier C	32	1,3783	1,4191	0.2%	0.463	1.02 [0.96–1.10]	0.491
	TT vs. CC	30	1,3628	1,3887	0.0%	0.495	1.00 [0.76–1.31]	0.991
	TT vs. CC+CT	30	1,3628	1,3887	0.0%	0.512	1.00 [0.76–1.31]	0.985
	CT vs. CC	32	1,3783	1,4191	17.7%	0.190	1.03 [0.95–1.12]	0.472
	CT+TT vs. CC	32	1,3783	1,4191	24.2%	0.110	1.03 [0.95–1.12]	0.446
African	Allele T vs. allele C	3	246	343	0.0%	0.517	3.66 [2.34–5.71]	<0.001
	Carrier T vs. carrier C	3	246	343	0.0%	0.666	3.08 [1.91–4.96]	<0.001
	TT vs. CC	2	192	269	0.0%	0.885	6.38 [1.53–26.56]	0.011
	TT vs. CC+CT	2	192	269	0.0%	0.986	4.83 [1.16–20.08]	0.030
	CT vs. CC	3	246	343	0.0%	0.437	3.77 [2.27–6.28]	<0.001
	CT+TT vs. CC	3	246	343	0.0%	0.434	4.02 [2.46–6.57]	<0.001


*N, number; *P*-heterogeneity, *P-*value of heterogeneity test; OR, odds ratio; CI, confidence interval; *P*-association, *P-*value of association test.*

Next, we performed subgroup analyses based upon control source (Supplementary Table S4) and cancer type (Supplementary Table S5 and Supplementary Figures S7–S10), targeting the Caucasian population. Similar positive results were detected in the “Head and neck cancer” subgroup analysis (Supplementary Table S5). Even though no significant associations were found in the “PB” or “HB” subgroup analyses (Supplementary Table S4, all *P*-association > 0.05), there was a positive association between *GSTP1* rs1138272 and the risk of colorectal cancer in the Caucasian population for the models of TT vs. CC (Supplementary Table S5, *P*-association = 0.52, OR = 1.21) and TT vs. CC+CT (*P*-association = 0.023, OR = 0.52). With regard to “Lung cancer,” we observed a slightly increased risk in the Caucasian population under the models of allele T vs. allele C (*P*-association = 0.015, OR = 1.21), carrier T vs. carrier C (*P*-association = 0.044, OR = 1.18), CT vs. CC (*P*-association = 0.032, OR = 1.20), and CT+TT vs. CC (*P*-association = 0.020, OR = 1.22). Further, to eliminate the effects of the HB controls in the results of the Caucasian population, we also performed another subgroup analysis based upon cancer type using the Caucasian cases and population-based negative controls. Similar results were detected for colorectal and lung cancer (Supplementary Table S6). These data revealed that the TT genotype of the *GSTP1* rs1138272 polymorphism may decrease susceptibility to “Colorectal” cancers but increase susceptibility to “Head and neck” cancers, while the CT genotype may be associated with lung cancer risk in the Caucasian population.

### Publication Bias and Sensitivity Analysis Results

**FIGURE 4:**
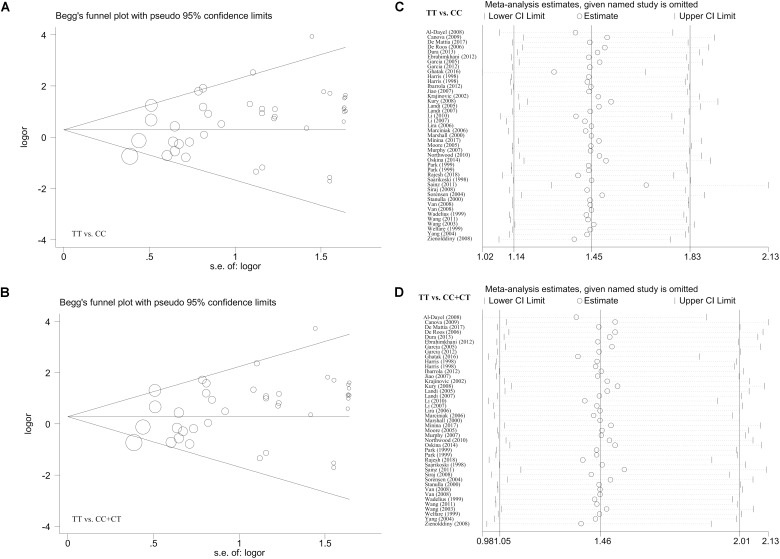
The publication bias plot according to the Begg’s test and the sensitivity analysis data for the overall population under the models of TT vs. CC and TT vs. CC+CT. **(A,B)** Begg’s test; **(C,D)** sensitivity analysis.

In the meta-analysis of the overall population, the Begg’s and Egger’s tests revealed (Supplementary Table S7) a presence of potential publication bias for the allele model in the Begg’s test (*P*-Begg’s test = 0.028) or the allele (*P*-Egger = 0.013), carrier (*P*-Egger = 0.035), homozygote (*P*-Egger = 0.013), recessive (*P*-Egger = 0.013), and dominant (*P*-Egger = 0.046) models in the Egger’s test. For the analysis targeting the Caucasian population (Supplementary Table S8), we only detected potential publication bias for the homozygote and recessive models (*P*-Egger < 0.05; *P*-Egger < 0.05). However, this slight publication bias only existed for the homozygote (*P*-Egger = 0.049) and recessive (*P*-Egger = 0.044) models using the Caucasian cases and population-based negative controls in the Egger’s test (Supplementary Table S9). Figures 4A,B and Supplementary Figures S11A,B, S12A,B present the relative publication bias plots according to the Begg’s tests as examples.

Additionally, we did not observe any remarkable alteration of the summary OR and corresponding 95% CI value when the individual case-control studies were removed one by one in our sensitivity analysis, confirming the abovementioned stability of the results. Some of the sensitivity analysis data (Figures 4C,D and Supplementary Figures S11C,D, S12C,D) are shown as examples.

## Discussion

The *GSTP1* rs1138272 polymorphism may be related to the risk of non-small cell lung cancer in the Norwegian population (Zienolddiny et al., 2008) and lung cancer in the Caucasian population of the United States (Wang et al., 2003). Nevertheless, no association was found between this polymorphism and lung cancer in Denmark (Sorensen et al., 2004) or with lung cancer in individuals in Russia who smoke (Minina et al., 2017). Hence, comprehensive analyses via the meta-analysis approach are meaningful.

In 2013, 28 case-control studies including 26 articles (Harris et al., 1998; Saarikoski et al., 1998; Wadelius et al., 1999; Wang et al., 2003, 2011; Barnette et al., 2004; Sorensen et al., 2004; Yang et al., 2004; Garcia-Closas et al., 2005; Landi et al., 2005, 2007; Moore et al., 2005; Marciniak et al., 2006; Jiao et al., 2007; Kim et al., 2007; Murphy et al., 2007; Al-Dayel et al., 2008; Kury et al., 2008; Siraj et al., 2008; Van Emburgh et al., 2008; Zienolddiny et al., 2008; Canova et al., 2009; Marie-Genica Consortium on Genetic Susceptibility for Menopausal Hormone Therapy Related Breast Cancer Risk, 2010; Northwood et al., 2010; Ebrahimkhani et al., 2012; Ibarrola-Villava et al., 2012) were recruited into a meta-analysis performed by Huang et al. (2013). The results indicated that the *GSTP1* rs1138272 polymorphism appears to be associated with an increased risk of cancer, particularly lung cancer in the Asian population (Huang et al., 2013). In our analysis, we collected the available published articles as thoroughly as possible through a systematic search of five online electronic databases. The included case-control studies that were selected using our strict inclusion and exclusion criteria. We removed one case-control study in which the data were not in HWE (Marie-Genica Consortium on Genetic Susceptibility for Menopausal Hormone Therapy Related Breast Cancer Risk, 2010), and we removed two additional studies (Barnette et al., 2004) because they failed to meet the requirement of reporting the genotype frequency in both the case and control group. Moreover, 17 new articles (Park et al., 1999; Welfare et al., 1999; Marshall et al., 2000; Stanulla et al., 2000; Krajinovic et al., 2002; De Roos et al., 2006; Lira et al., 2006; Li et al., 2007, 2010; Sainz et al., 2011; Garcia-Gonzalez et al., 2012; Dura et al., 2013; Oskina et al., 2014; Ghatak et al., 2016; De Mattia et al., 2017; Minina et al., 2017; Rajesh et al., 2018) were added. Finally, a total of 40 articles were included in our updated meta-analysis. After the data extraction, 43 case-control studies were enrolled in the meta-analysis under the allele, heterozygote, dominant, and carrier genetic models. All of the studies follow Hardy–Weinberg equilibrium and exhibit high quality. Three studies (Marshall et al., 2000; Lira et al., 2006; Van Emburgh et al., 2008) were excluded in the homozygote and recessive models because the CC genotype frequency in both the case and control group was equal to zero. We detected a potential correlation between the TT genotype of *GSTP1* rs1138272 and cancer susceptibility in the Asian population, which is partly in agreement with the previously reported data (Huang et al., 2013). In addition, we found that the *GSTP1* rs1138272 polymorphism may be associated with an increased risk of cancer in the African population.

Ye et al. (2006) recruited four case-control studies (Harris et al., 1998; Wang et al., 2003; Sorensen et al., 2004; Yang et al., 2004) to conduct a meta-analysis on the association between *GSTP1* rs1138272 and lung cancer risk (Ye et al., 2006). This group did not provide evidence for a strong association between *GSTP1* rs1138272 and lung cancer susceptibility (Ye et al., 2006). Yan et al. (2016) included five case-control studies (Harris et al., 1998; Wang et al., 2003; Yang et al., 2004; Zienolddiny et al., 2008; Vural et al., 2012) to perform another relative meta-analysis (Yan et al., 2016) wherein an association between *GSTP1* rs1138272 and increased lung cancer risk was detected (Yan et al., 2016). Here, in our subgroup analysis of lung cancer, we removed one study that was not in HWE (Vural et al., 2012) and added two case-control studies (Saarikoski et al., 1998; Minina et al., 2017) for the pooled analysis. Based on the available data within seven articles (Harris et al., 1998; Saarikoski et al., 1998; Wang et al., 2003; Sorensen et al., 2004; Yang et al., 2004; Zienolddiny et al., 2008; Minina et al., 2017), we failed to detect a relationship between *GSTP1* rs1138272 and lung cancer risk in the overall population. However, when we enrolled the Caucasian cases and population-based negative controls in six studies (Harris et al., 1998; Saarikoski et al., 1998; Wang et al., 2003; Sorensen et al., 2004; Zienolddiny et al., 2008; Minina et al., 2017) to perform another subgroup analysis by cancer type, we found that the CT genotype of *GSTP1* rs1138272 may confer the highest susceptibility to the lung cancer in the Caucasian population.

Previously, three meta-analyses of data on brain tumors were published (Lai et al., 2005; Fan et al., 2013; Geng et al., 2016). Each of these meta-analyses included four case-control studies (Ezer et al., 2002; De Roos et al., 2003; Wrensch et al., 2004; Schwartzbaum et al., 2007). Unfortunately, based on our screening strategy, these studies could not be enrolled in our comprehensive analyses. One of the studies was not in HWE (Ezer et al., 2002), and the others failed to provide the complete genotype frequency of CT and CT within *GSTP1* rs1138272 (De Roos et al., 2003; Wrensch et al., 2004; Schwartzbaum et al., 2007).

With regard to colorectal cancer, Li et al. (2013) performed a relevant meta-analysis including seven case-control studies (3,173 cases/3,323 controls) (Welfare et al., 1999; Sachse et al., 2002; Landi et al., 2005; Kury et al., 2008; Sainz et al., 2011; Wang et al., 2011; Ebrahimkhani et al., 2012) in 2013 and reported a negative association between *GSTP1* rs1138272 and colorectal cancer risk (Li et al., 2013). Herein, we ruled out one the studies included by Li et al. (2013) because it deviated from Hardy–Weinberg equilibrium (Sachse et al., 2002), and we included three new eligible studies (Harris et al., 1998; Moore et al., 2005; Northwood et al., 2010) to perform an updated analysis. Compared with the “colorectal cancer” subgroup of Huang et al. (2013), two case-control studies (Welfare et al., 1999; Sainz et al., 2011) were added. Despite the additional studies, a similar negative conclusion in the overall population was observed in our updated meta-analysis. However, when targeting the Caucasian population, we found that the TT genotype of *GSTP1* rs1138272 may be positively linked to a decreased risk of colorectal cancer in Caucasians. Some environmental factors, such as nutrition and other exposures, may serve as the potential contributory reasons for the observed differences of susceptibility in different populations or cancers.

Although the results of the sensitivity analysis indicated the stability of the data, our study is not without several limitations. The issue of small sample sizes should be considered fully when interpreting certain results. For example, an elevated cancer risk was observed for the “African” subgroup under all of the genetic models; however, only two case-control studies were included for the homozygote and recessive models. Due to the lack of data, we had to consider all cancers together for the Asian and African populations. Despite the positive conclusions obtained, more case-control studies in the Asian and African population are warranted to enable more accurate cancer type-specific subgroup analyses.

Although we observed a potential relationship between *GSTP1* rs1138272 and the risk of colorectal, lung, head and neck cancers within Caucasians, no more than 10 case-control studies were enrolled, and more detailed head and neck cancer types were not evaluated due to the lack of sufficient data. Furthermore, the role of *GSTP1* rs1138272 in other cancer types has not yet been investigated. Only one case-control study was available for the stratified analysis of bladder, liver, or pancreatic cancer.

In addition, a high degree of inter-study heterogeneity and potential publication bias were observed in certain comparisons. The level of heterogeneity and publication bias was reduced in the analyses of the Caucasian population, suggesting that the “ethnicity” factor is essential for the assessment of the distinct role of *GSTP1* rs1138272 in cancer risk.

Considering the role of possible linkage disequilibrium in the genetic susceptibility to different cancers, we tried to extract the data of *GSTP1* haplotypes in the enrolled case-control studies. Nevertheless, not enough relevant data supported the performance of pooling analysis. In addition, the *GSTP1* rs1138272 polymorphism together with the *GSTM1* (glutathione *S*-transferase M1) null genotype was reported to be associated with the risk of colon or rectal cancer in the Indian population (Wang et al., 2011). The limited availability of useable data also prevented us from exploring the genetic effects of the *GSTP1* polymorphism combined with variants of other genes in specific cancer types. The factors, such as the age of onset, sex, lifestyle, environmental exposure, cancer source, linkage disequilibrium, synergistic interaction between genes, etiologies, relapses, and other patient clinical characterizations should be considered carefully when more data is available.

Above all, our pooled analysis consisting of the currently available eligible case-control studies demonstrated that the *GSTP1* rs1138272 polymorphism is associated with the susceptibility to overall cancer in the Asian and African populations and, moreover, this polymorphism may be linked to the risk of colorectal, lung or head and neck cancers in the Caucasian population. More eligible case-control studies containing cases with distinct cancers in various ethnic backgrounds are necessary for a more precise and relatively objective estimation.

## Author Contributions

FD, J-PL, and YZ were conducted by database searching and screening process. FD, G-HQ, and Z-CS was performed by statistical analysis. FD and J-PL wrote the manuscript. Y-HY reviewed the manuscript. All authors approved the final version of the manuscript.

## Conflict of Interest Statement

The authors declare that the research was conducted in the absence of any commercial or financial relationships that could be construed as a potential conflict of interest.

## Supplementary Material

FIGURE S1 Forest plot of the meta-analysis in the overall population (allele T vs. allele C model).

FIGURE S2 Forest plot of the meta-analysis in the overall population (carrier T vs. carrier C model).

FIGURE S3 Forest plot of the meta-analysis in the overall population (CT vs. CC model).

FIGURE S4 Forest plot of the meta-analysis in the overall population (CT+TT vs. CC model).

FIGURE S5 Forest plot of the subgroup analysis by control source in the overall population (TT vs. CC model).

FIGURE S6 Forest plot of the subgroup analysis by cancer type in the overall population (the allele T vs. allele C model).

FIGURE S7 Forest plot of the subgroup analysis by cancer type in the Caucasian population (TT vs. CC model).

FIGURE S8 Forest plot of the subgroup analysis by cancer type in the Caucasian population (TT vs. CC+CT model).

FIGURE S9 Forest plot of the subgroup analysis by cancer type in the Caucasian population (CT vs. CC model).

FIGURE S10 Forest plot of the subgroup analysis by cancer type in the Caucasian population (CT+TT vs. CC model).

FIGURE S11 The publication bias plot according to the Begg’s test and sensitivity analysis data for the Caucasian population under the models of TT vs. CC and TT vs. CC+CT. **(A,B)** Begg’s test; **(C,D)** sensitivity analysis.

FIGURE S12 The publication bias plot according to the Begg’s test and sensitivity analysis data for the Caucasian population under the models of CT vs. CC and CT+TT vs. CC. **(A,B)** Begg’s test; **(C,D)** sensitivity analysis.

TABLE S1 Database searching terms (up to September 2018).

TABLE S2 Subgroup analysis by control source in the overall population.

TABLE S3 Subgroup analysis by cancer type in the overall population.

TABLE S4 Subgroup analysis by control source in the Caucasian population.

TABLE S5 Subgroup analysis by cancer type in the Caucasian population.

TABLE S6 Subgroup analysis by cancer type using the Caucasian cases and population-based negative controls.

TABLE S7 Publication bias analysis in the overall population.

TABLE S8 Publication bias analysis in the Caucasian population.

TABLE S9 Publication bias analysis in the Caucasian cases and population-based negative controls.
