# Supplementary material for: Comprehensive Analysis of the Association Between the rs1138272 Polymorphism of the GSTP1 Gene and Cancer Susceptibility
Source: Front Physiol. 2019 Jan 25;9:1897. doi: 10.3389/fphys.2018.01897 (PMC6355699; doi:10.3389/fphys.2018.01897)
Supplement: FIGURE S1 — Forest plot of the meta-analysis in the overall population (allele T vs. allele C model). [file Data_Sheet_1.PDF]

Fig. S1

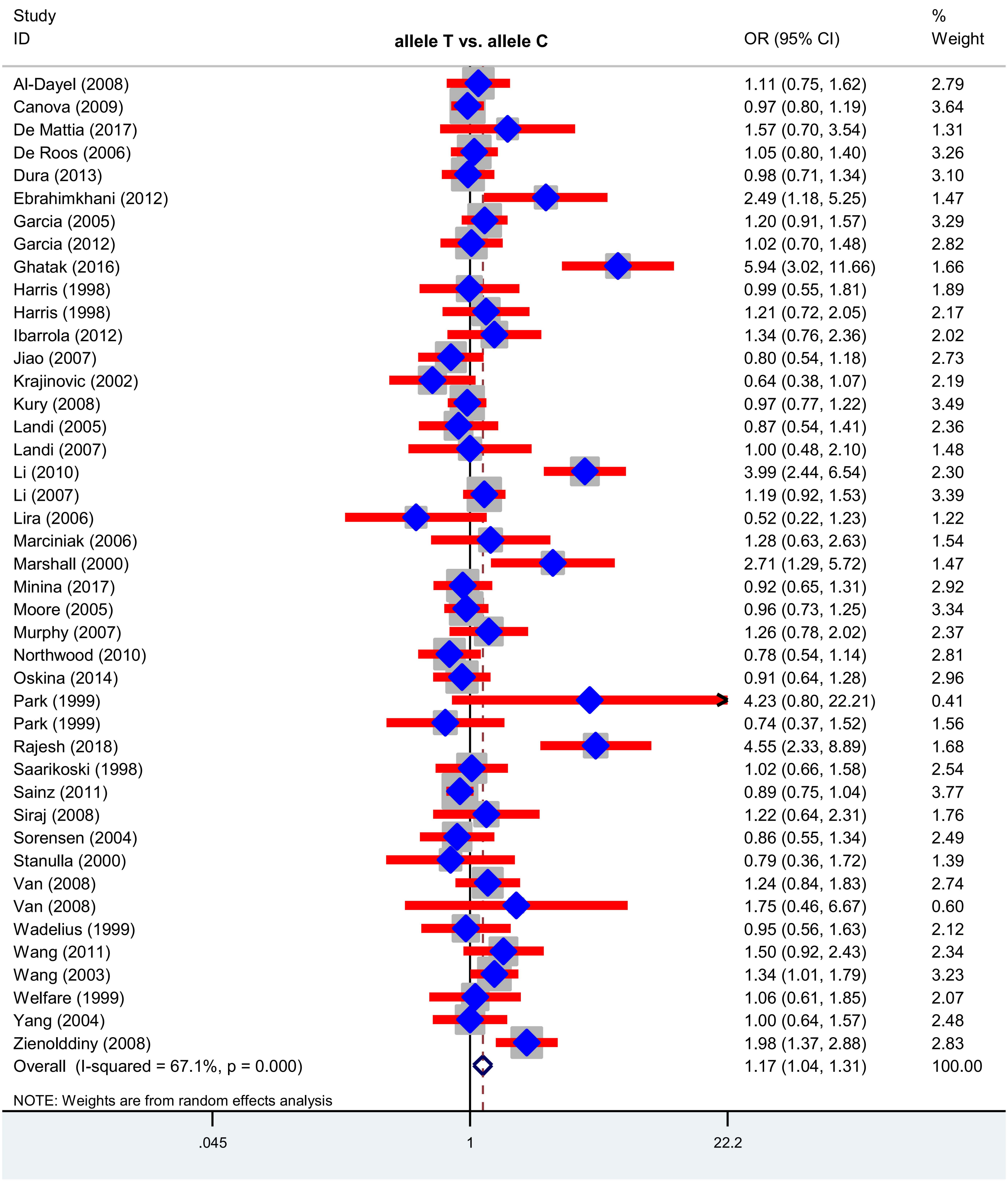
Study
ID
allele T vs. allele C
OR (95% CI)
%
Weight
Al-Dayel (2008) 1.11 (0.75, 1.62) 2.79
Canova (2009) 0.97 (0.80, 1.19) 3.64
De Mattia (2017) 1.57 (0.70, 3.54) 1.31
De Roos (2006) 1.05 (0.80, 1.40) 3.26
Dura (2013) 0.98 (0.71, 1.34) 3.10
Ebrahimkhani (2012) 2.49 (1.18, 5.25) 1.47
Garcia (2005) 1.20 (0.91, 1.57) 3.29
Garcia (2012) 1.02 (0.70, 1.48) 2.82
Ghatak (2016) 5.94 (3.02, 11.66) 1.66
Harris (1998) 0.99 (0.55, 1.81) 1.89
Harris (1998) 1.21 (0.72, 2.05) 2.17
Ibarrola (2012) 1.34 (0.76, 2.36) 2.02
Jiao (2007) 0.80 (0.54, 1.18) 2.73
Krajinovic (2002) 0.64 (0.38, 1.07) 2.19
Kury (2008) 0.97 (0.77, 1.22) 3.49
Landi (2005) 0.87 (0.54, 1.41) 2.36
Landi (2007) 1.00 (0.48, 2.10) 1.48
Li (2010) 3.99 (2.44, 6.54) 2.30
Li (2007) 1.19 (0.92, 1.53) 3.39
Lira (2006) 0.52 (0.22, 1.23) 1.22
Marciniak (2006) 1.28 (0.63, 2.63) 1.54
Marshall (2000) 2.71 (1.29, 5.72) 1.47
Minina (2017) 0.92 (0.65, 1.31) 2.92
Moore (2005) 0.96 (0.73, 1.25) 3.34
Murphy (2007) 1.26 (0.78, 2.02) 2.37
Northwood (2010) 0.78 (0.54, 1.14) 2.81
Oskina (2014) 0.91 (0.64, 1.28) 2.96
Park (1999) 4.23 (0.80, 22.21) 0.41
Park (1999) 0.74 (0.37, 1.52) 1.56
Rajesh (2018) 4.55 (2.33, 8.89) 1.68
Saarikoski (1998) 1.02 (0.66, 1.58) 2.54
Sainz (2011) 0.89 (0.75, 1.04) 3.77
Siraj (2008) 1.22 (0.64, 2.31) 1.76
Sorensen (2004) 0.86 (0.55, 1.34) 2.49
Stanulla (2000) 0.79 (0.36, 1.72) 1.39
Van (2008) 1.24 (0.84, 1.83) 2.74
Van (2008) 1.75 (0.46, 6.67) 0.60
Wadelius (1999) 0.95 (0.56, 1.63) 2.12
Wang (2011) 1.50 (0.92, 2.43) 2.34
Wang (2003) 1.34 (1.01, 1.79) 3.23
Welfare (1999) 1.06 (0.61, 1.85) 2.07
Yang (2004) 1.00 (0.64, 1.57) 2.48
Zienolddiny (2008) 1.98 (1.37, 2.88) 2.83
Overall (I-squared = 67.1%, p = 0.000) 1.17 (1.04, 1.31) 100.00
NOTE: Weights are from random effects analysis
.045
1
22.2

Fig. S2

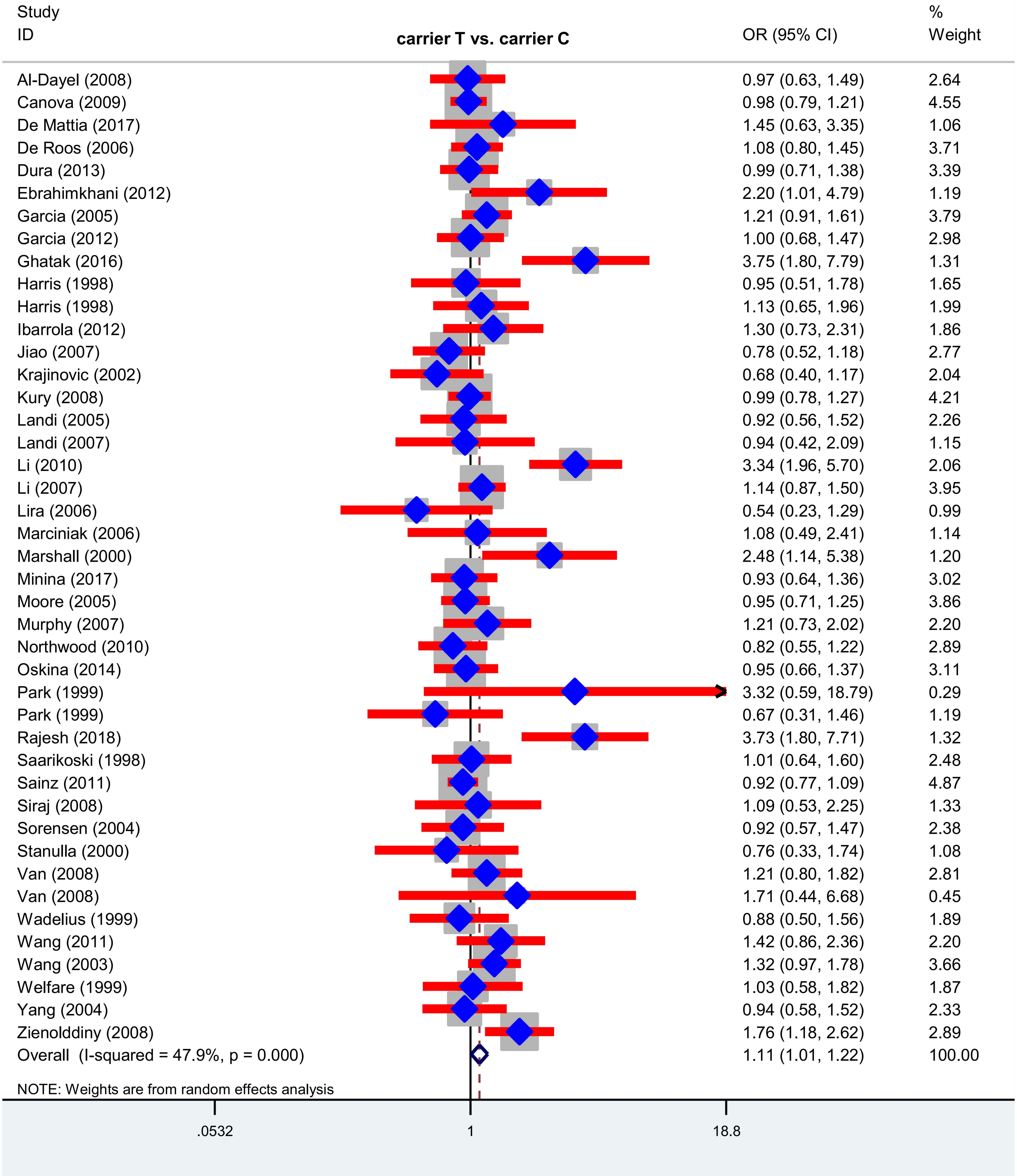
Study
ID
carrier T vs. carrier C
OR (95% CI)
%
Weight
Al-Dayel (2008) 0.97 (0.63, 1.49) 2.64
Canova (2009) 0.98 (0.79, 1.21) 4.55
De Mattia (2017) 1.45 (0.63, 3.35) 1.06
De Roos (2006) 1.08 (0.80, 1.45) 3.71
Dura (2013) 0.99 (0.71, 1.38) 3.39
Ebrahimkhani (2012) 2.20 (1.01, 4.79) 1.19
Garcia (2005) 1.21 (0.91, 1.61) 3.79
Garcia (2012) 1.00 (0.68, 1.47) 2.98
Ghatak (2016) 3.75 (1.80, 7.79) 1.31
Harris (1998) 0.95 (0.51, 1.78) 1.65
Harris (1998) 1.13 (0.65, 1.96) 1.99
Ibarrola (2012) 1.30 (0.73, 2.31) 1.86
Jiao (2007) 0.78 (0.52, 1.18) 2.77
Krajinovic (2002) 0.68 (0.40, 1.17) 2.04
Kury (2008) 0.99 (0.78, 1.27) 4.21
Landi (2005) 0.92 (0.56, 1.52) 2.26
Landi (2007) 0.94 (0.42, 2.09) 1.15
Li (2010) 3.34 (1.96, 5.70) 2.06
Li (2007) 1.14 (0.87, 1.50) 3.95
Lira (2006) 0.54 (0.23, 1.29) 0.99
Marciniak (2006) 1.08 (0.49, 2.41) 1.14
Marshall (2000) 2.48 (1.14, 5.38) 1.20
Minina (2017) 0.93 (0.64, 1.36) 3.02
Moore (2005) 0.95 (0.71, 1.25) 3.86
Murphy (2007) 1.21 (0.73, 2.02) 2.20
Northwood (2010) 0.82 (0.55, 1.22) 2.89
Oskina (2014) 0.95 (0.66, 1.37) 3.11
Park (1999) 3.32 (0.59, 18.79) 0.29
Park (1999) 0.67 (0.31, 1.46) 1.19
Rajesh (2018) 3.73 (1.80, 7.71) 1.32
Saarikoski (1998) 1.01 (0.64, 1.60) 2.48
Sainz (2011) 0.92 (0.77, 1.09) 4.87
Siraj (2008) 1.09 (0.53, 2.25) 1.33
Sorensen (2004) 0.92 (0.57, 1.47) 2.38
Stanulla (2000) 0.76 (0.33, 1.74) 1.08
Van (2008) 1.21 (0.80, 1.82) 2.81
Van (2008) 1.71 (0.44, 6.68) 0.45
Wadelius (1999) 0.88 (0.50, 1.56) 1.89
Wang (2011) 1.42 (0.86, 2.36) 2.20
Wang (2003) 1.32 (0.97, 1.78) 3.66
Welfare (1999) 1.03 (0.58, 1.82) 1.87
Yang (2004) 0.94 (0.58, 1.52) 2.33
Zienolddiny (2008) 1.76 (1.18, 2.62) 2.89
Overall (I-squared = 47.9%, p = 0.000) 1.11 (1.01, 1.22) 100.00
NOTE: Weights are from random effects analysis
.0532
1
18.8

Fig. S3

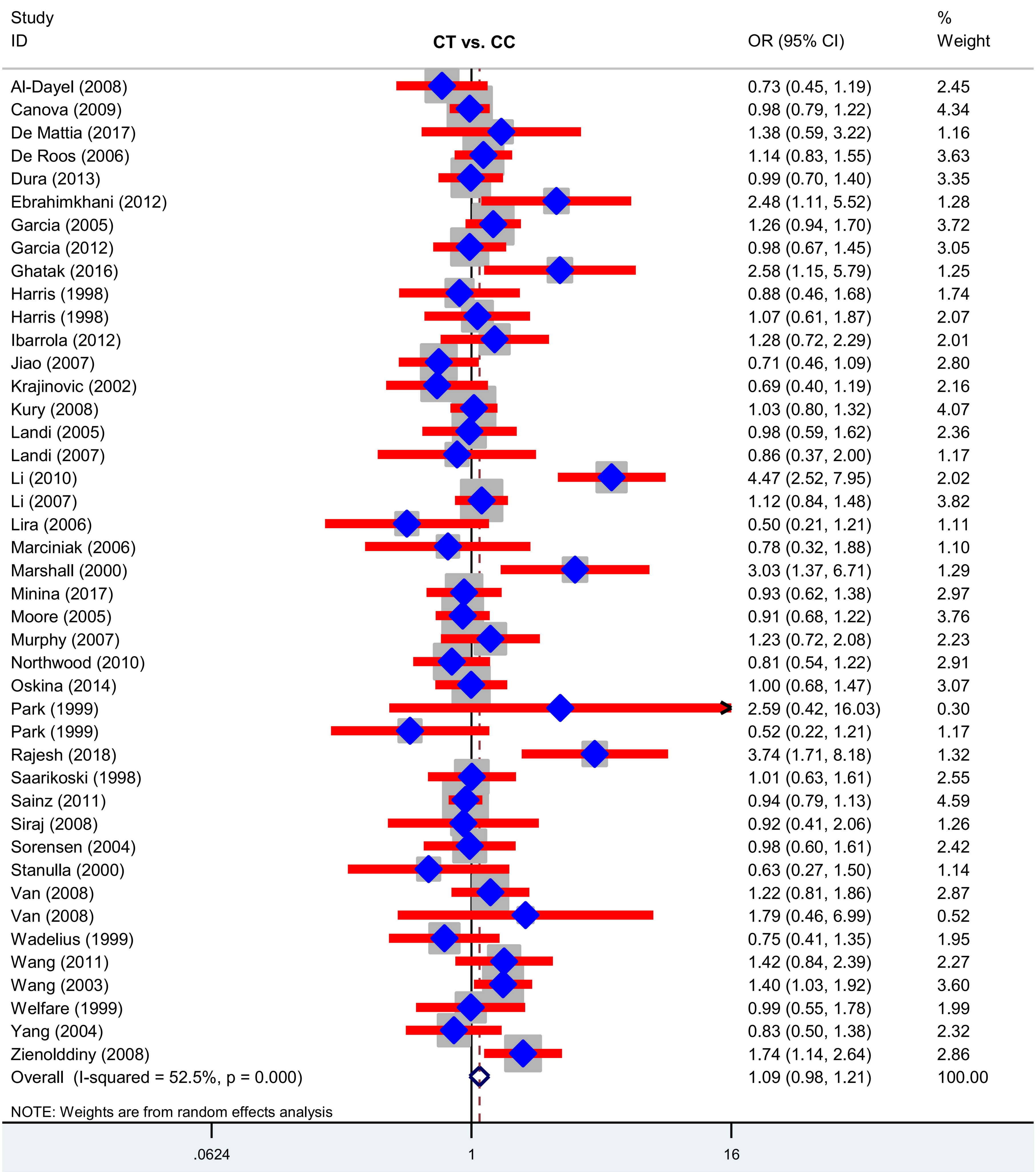
Study
ID
CT vs. CC
OR (95% CI)
%
Weight
Al-Dayel (2008) 0.73 (0.45, 1.19) 2.45
Canova (2009) 0.98 (0.79, 1.22) 4.34
De Mattia (2017) 1.38 (0.59, 3.22) 1.16
De Roos (2006) 1.14 (0.83, 1.55) 3.63
Dura (2013) 0.99 (0.70, 1.40) 3.35
Ebrahimkhani (2012) 2.48 (1.11, 5.52) 1.28
Garcia (2005) 1.26 (0.94, 1.70) 3.72
Garcia (2012) 0.98 (0.67, 1.45) 3.05
Ghatak (2016) 2.58 (1.15, 5.79) 1.25
Harris (1998) 0.88 (0.46, 1.68) 1.74
Harris (1998) 1.07 (0.61, 1.87) 2.07
Ibarrola (2012) 1.28 (0.72, 2.29) 2.01
Jiao (2007) 0.71 (0.46, 1.09) 2.80
Krajinovic (2002) 0.69 (0.40, 1.19) 2.16
Kury (2008) 1.03 (0.80, 1.32) 4.07
Landi (2005) 0.98 (0.59, 1.62) 2.36
Landi (2007) 0.86 (0.37, 2.00) 1.17
Li (2010) 4.47 (2.52, 7.95) 2.02
Li (2007) 1.12 (0.84, 1.48) 3.82
Lira (2006) 0.50 (0.21, 1.21) 1.11
Marciniak (2006) 0.78 (0.32, 1.88) 1.10
Marshall (2000) 3.03 (1.37, 6.71) 1.29
Minina (2017) 0.93 (0.62, 1.38) 2.97
Moore (2005) 0.91 (0.68, 1.22) 3.76
Murphy (2007) 1.23 (0.72, 2.08) 2.23
Northwood (2010) 0.81 (0.54, 1.22) 2.91
Oskina (2014) 1.00 (0.68, 1.47) 3.07
Park (1999) 2.59 (0.42, 16.03) 0.30
Park (1999) 0.52 (0.22, 1.21) 1.17
Rajesh (2018) 3.74 (1.71, 8.18) 1.32
Saarikoski (1998) 1.01 (0.63, 1.61) 2.55
Sainz (2011) 0.94 (0.79, 1.13) 4.59
Siraj (2008) 0.92 (0.41, 2.06) 1.26
Sorensen (2004) 0.98 (0.60, 1.61) 2.42
Stanulla (2000) 0.63 (0.27, 1.50) 1.14
Van (2008) 1.22 (0.81, 1.86) 2.87
Van (2008) 1.79 (0.46, 6.99) 0.52
Wadelius (1999) 0.75 (0.41, 1.35) 1.95
Wang (2011) 1.42 (0.84, 2.39) 2.27
Wang (2003) 1.40 (1.03, 1.92) 3.60
Welfare (1999) 0.99 (0.55, 1.78) 1.99
Yang (2004) 0.83 (0.50, 1.38) 2.32
Zienolddiny (2008) 1.74 (1.14, 2.64) 2.86
Overall (I-squared = 52.5%, p = 0.000) 1.09 (0.98, 1.21) 100.00
NOTE: Weights are from random effects analysis
.0624
1
16

Fig. S4

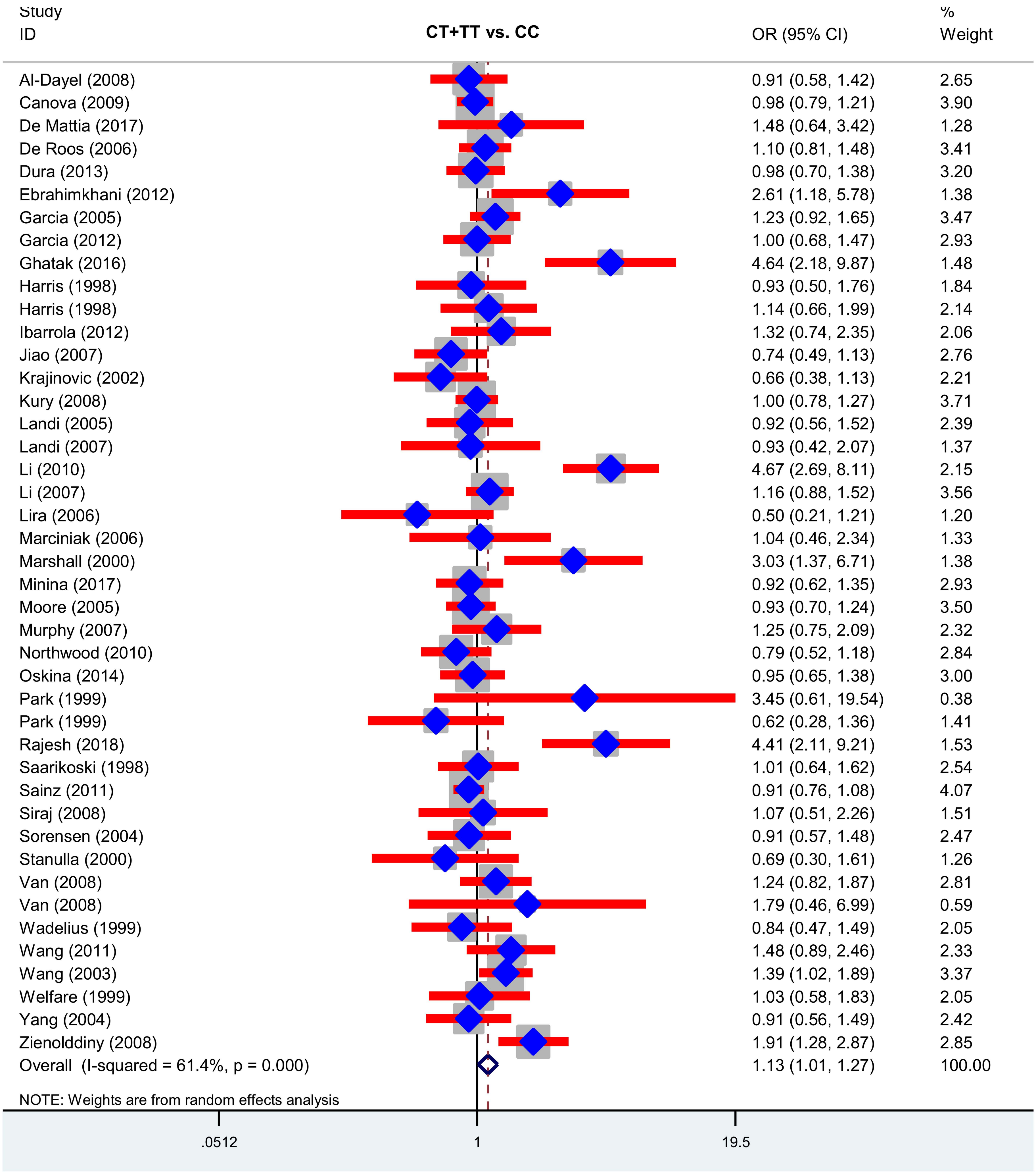

Study
ID
CT+TT vs. CC
OR (95% CI)
%
Weight
Al-Dayel (2008) 0.91 (0.58, 1.42) 2.65
Canova (2009) 0.98 (0.79, 1.21) 3.90
De Mattia (2017) 1.48 (0.64, 3.42) 1.28
De Roos (2006) 1.10 (0.81, 1.48) 3.41
Dura (2013) 0.98 (0.70, 1.38) 3.20
Ebrahimkhani (2012) 2.61 (1.18, 5.78) 1.38
Garcia (2005) 1.23 (0.92, 1.65) 3.47
Garcia (2012) 1.00 (0.68, 1.47) 2.93
Ghatak (2016) 4.64 (2.18, 9.87) 1.48
Harris (1998) 0.93 (0.50, 1.76) 1.84
Harris (1998) 1.14 (0.66, 1.99) 2.14
Ibarrola (2012) 1.32 (0.74, 2.35) 2.06
Jiao (2007) 0.74 (0.49, 1.13) 2.76
Krajinovic (2002) 0.66 (0.38, 1.13) 2.21
Kury (2008) 1.00 (0.78, 1.27) 3.71
Landi (2005) 0.92 (0.56, 1.52) 2.39
Landi (2007) 0.93 (0.42, 2.07) 1.37
Li (2010) 4.67 (2.69, 8.11) 2.15
Li (2007) 1.16 (0.88, 1.52) 3.56
Lira (2006) 0.50 (0.21, 1.21) 1.20
Marciniak (2006) 1.04 (0.46, 2.34) 1.33
Marshall (2000) 3.03 (1.37, 6.71) 1.38
Minina (2017) 0.92 (0.62, 1.35) 2.93
Moore (2005) 0.93 (0.70, 1.24) 3.50
Murphy (2007) 1.25 (0.75, 2.09) 2.32
Northwood (2010) 0.79 (0.52, 1.18) 2.84
Oskina (2014) 0.95 (0.65, 1.38) 3.00
Park (1999) 3.45 (0.61, 19.54) 0.38
Park (1999) 0.62 (0.28, 1.36) 1.41
Rajesh (2018) 4.41 (2.11, 9.21) 1.53
Saarikoski (1998) 1.01 (0.64, 1.62) 2.54
Sainz (2011) 0.91 (0.76, 1.08) 4.07
Siraj (2008) 1.07 (0.51, 2.26) 1.51
Sorensen (2004) 0.91 (0.57, 1.48) 2.47
Stanulla (2000) 0.69 (0.30, 1.61) 1.26
Van (2008) 1.24 (0.82, 1.87) 2.81
Van (2008) 1.79 (0.46, 6.99) 0.59
Wadelius (1999) 0.84 (0.47, 1.49) 2.05
Wang (2011) 1.48 (0.89, 2.46) 2.33
Wang (2003) 1.39 (1.02, 1.89) 3.37
Welfare (1999) 1.03 (0.58, 1.83) 2.05
Yang (2004) 0.91 (0.56, 1.49) 2.42
Zienolddiny (2008) 1.91 (1.28, 2.87) 2.85
Overall (I-squared = 61.4%, p = 0.000) 1.13 (1.01, 1.27) 100.00
NOTE: Weights are from random effects analysis
.0512
1
19.5

Fig. S5

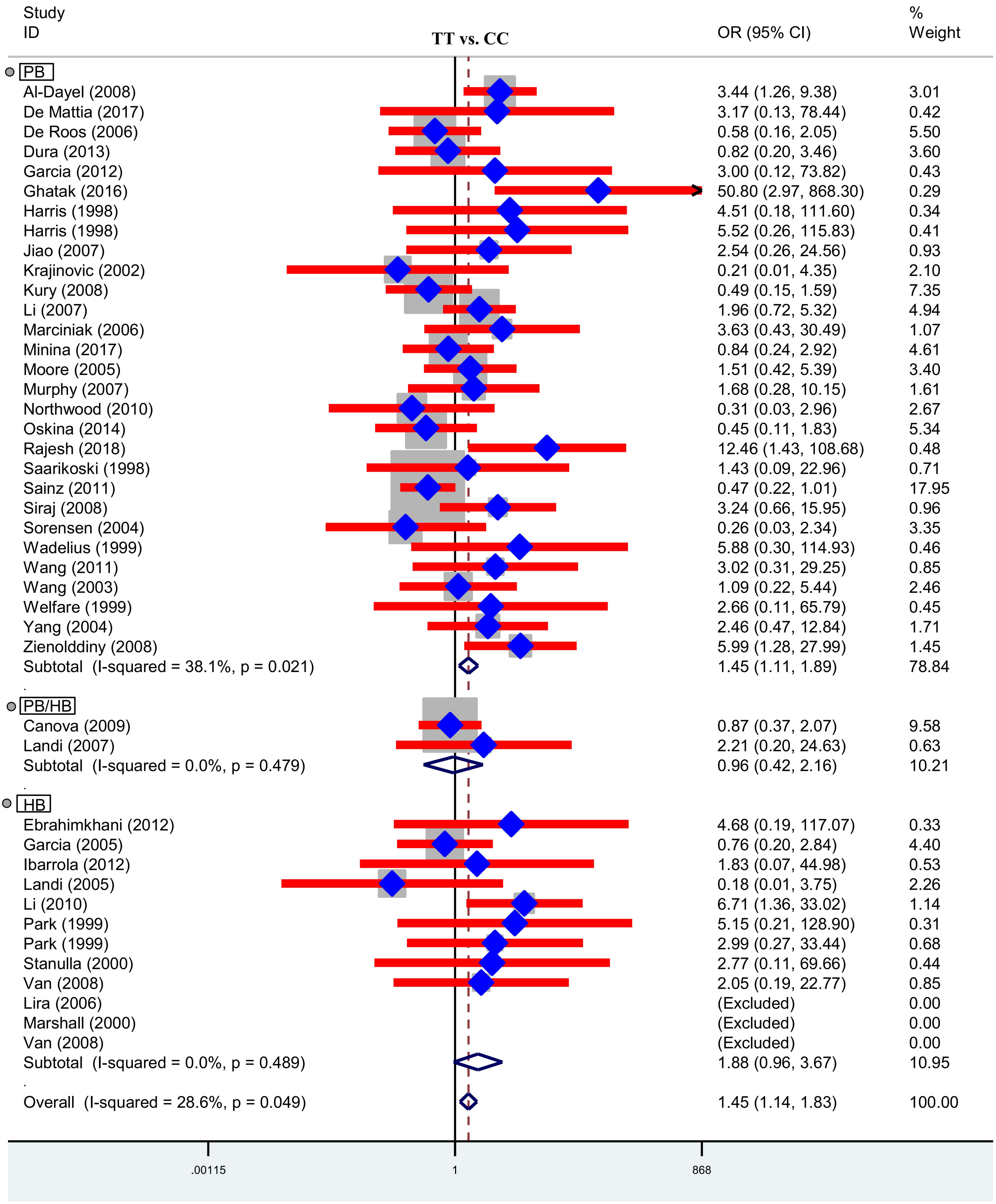

Study ID
TT vs. CC
OR (95% CI)
% Weight
PB
Al-Dayel (2008) 3.44 (1.26, 9.38) 3.01
De Mattia (2017) 3.17 (0.13, 78.44) 0.42
De Roos (2006) 0.58 (0.16, 2.05) 5.50
Dura (2013) 0.82 (0.20, 3.46) 3.60
Garcia (2012) 3.00 (0.12, 73.82) 0.43
Ghatak (2016) 50.80 (2.97, 868.30) 0.29
Harris (1998) 4.51 (0.18, 111.60) 0.34
Harris (1998) 5.52 (0.26, 115.83) 0.41
Jiao (2007) 2.54 (0.26, 24.56) 0.93
Krajinovic (2002) 0.21 (0.01, 4.35) 2.10
Kury (2008) 0.49 (0.15, 1.59) 7.35
Li (2007) 1.96 (0.72, 5.32) 4.94
Marciniak (2006) 3.63 (0.43, 30.49) 1.07
Minina (2017) 0.84 (0.24, 2.92) 4.61
Moore (2005) 1.51 (0.42, 5.39) 3.40
Murphy (2007) 1.68 (0.28, 10.15) 1.61
Northwood (2010) 0.31 (0.03, 2.96) 2.67
Oskina (2014) 0.45 (0.11, 1.83) 5.34
Rajesh (2018) 12.46 (1.43, 108.68) 0.48
Saarikoski (1998) 1.43 (0.09, 22.96) 0.71
Sainz (2011) 0.47 (0.22, 1.01) 17.95
Siraj (2008) 3.24 (0.66, 15.95) 0.96
Sorensen (2004) 0.26 (0.03, 2.34) 3.35
Wadelius (1999) 5.88 (0.30, 114.93) 0.46
Wang (2011) 3.02 (0.31, 29.25) 0.85
Wang (2003) 1.09 (0.22, 5.44) 2.46
Welfare (1999) 2.66 (0.11, 65.79) 0.45
Yang (2004) 2.46 (0.47, 12.84) 1.71
Zienolddiny (2008) 5.99 (1.28, 27.99) 1.45
Subtotal (I-squared = 38.1%, p = 0.021) 1.45 (1.11, 1.89) 78.84
.
PB/HB
Canova (2009) 0.87 (0.37, 2.07) 9.58
Landi (2007) 2.21 (0.20, 24.63) 0.63
Subtotal (I-squared = 0.0%, p = 0.479) 0.96 (0.42, 2.16) 10.21
.
HB
Ebrahimkhani (2012) 4.68 (0.19, 117.07) 0.33
Garcia (2005) 0.76 (0.20, 2.84) 4.40
Ibarrola (2012) 1.83 (0.07, 44.98) 0.53
Landi (2005) 0.18 (0.01, 3.75) 2.26
Li (2010) 6.71 (1.36, 33.02) 1.14
Park (1999) 5.15 (0.21, 128.90) 0.31
Park (1999) 2.99 (0.27, 33.44) 0.68
Stanulla (2000) 2.77 (0.11, 69.66) 0.44
Van (2008) 2.05 (0.19, 22.77) 0.85
Lira (2006) (Excluded) 0.00
Marshall (2000) (Excluded) 0.00
Van (2008) (Excluded) 0.00
Subtotal (I-squared = 0.0%, p = 0.489) 1.88 (0.96, 3.67) 10.95
.
Overall (I-squared = 28.6%, p = 0.049) 1.45 (1.14, 1.83) 100.00
.00115
1
868

Fig. S6

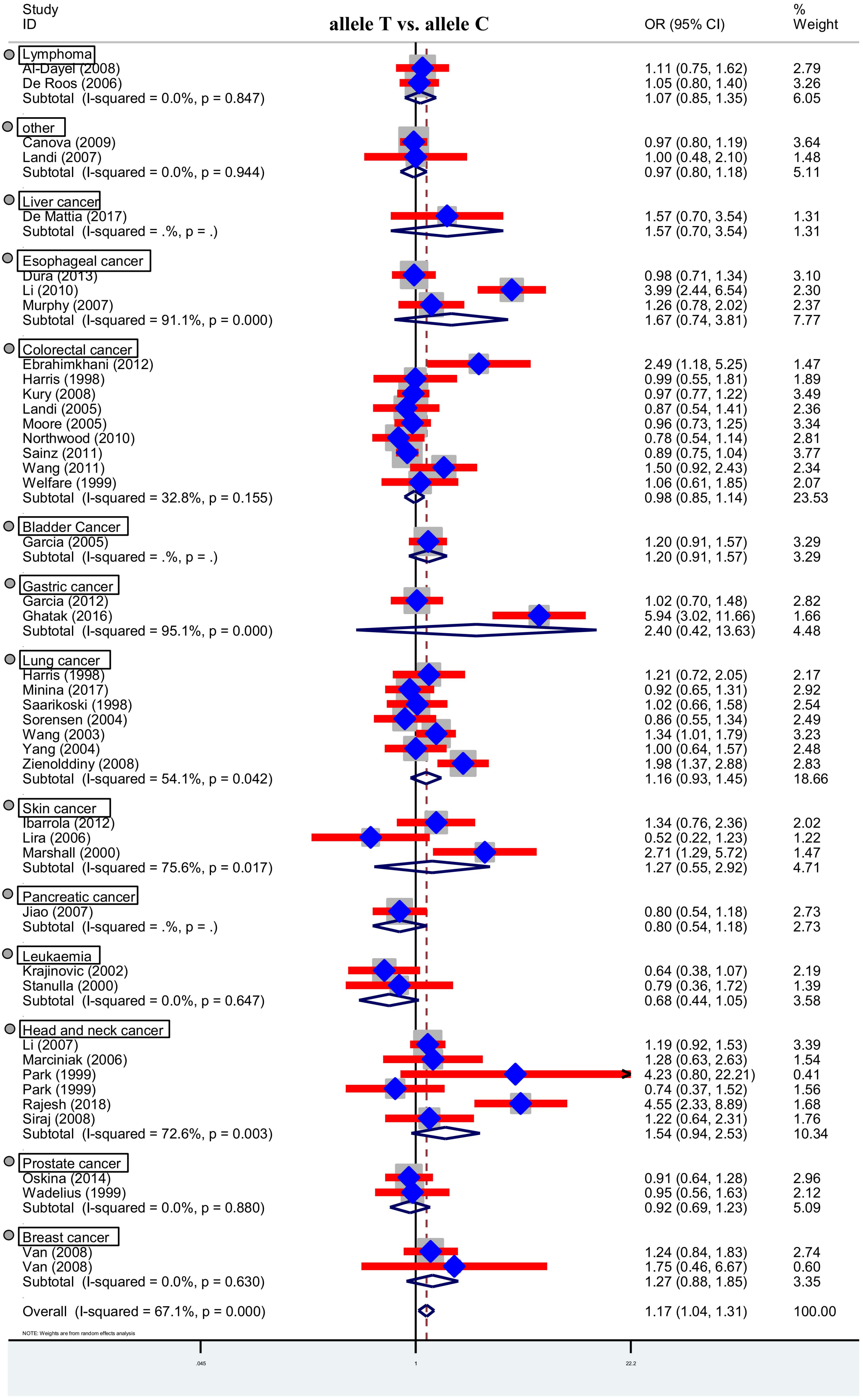

Study
ID
allele T vs. allele C
OR (95% CI)
%
Weight
Lymphoma
Al-Dayel (2008) 1.11 (0.75, 1.62) 2.79
De Roos (2006) 1.05 (0.80, 1.40) 3.26
Subtotal (I-squared = 0.0%, p = 0.847) 1.07 (0.85, 1.35) 6.05
other
Canova (2009) 0.97 (0.80, 1.19) 3.64
Landi (2007) 1.00 (0.48, 2.10) 1.48
Subtotal (I-squared = 0.0%, p = 0.944) 0.97 (0.80, 1.18) 5.11
Liver cancer
De Mattia (2017) 1.57 (0.70, 3.54) 1.31
Subtotal (I-squared = .%, p = .) 1.57 (0.70, 3.54) 1.31
Esophageal cancer
Dura (2013) 0.98 (0.71, 1.34) 3.10
Li (2010) 3.99 (2.44, 6.54) 2.30
Murphy (2007) 1.26 (0.78, 2.02) 2.37
Subtotal (I-squared = 91.1%, p = 0.000) 1.67 (0.74, 3.81) 7.77
Colorectal cancer
Ebrahimkhani (2012) 2.49 (1.18, 5.25) 1.47
Harris (1998) 0.99 (0.55, 1.81) 1.89
Kury (2008) 0.97 (0.77, 1.22) 3.49
Landi (2005) 0.87 (0.54, 1.41) 2.36
Moore (2005) 0.96 (0.73, 1.25) 3.34
Northwood (2010) 0.78 (0.54, 1.14) 2.81
Sainz (2011) 0.89 (0.75, 1.04) 3.77
Wang (2011) 1.50 (0.92, 2.43) 2.34
Welfare (1999) 1.06 (0.61, 1.85) 2.07
Subtotal (I-squared = 32.8%, p = 0.155) 0.98 (0.85, 1.14) 23.53
Bladder Cancer
Garcia (2005) 1.20 (0.91, 1.57) 3.29
Subtotal (I-squared = .%, p = .) 1.20 (0.91, 1.57) 3.29
Gastric cancer
Garcia (2012) 1.02 (0.70, 1.48) 2.82
Ghatak (2016) 5.94 (3.02, 11.66) 1.66
Subtotal (I-squared = 95.1%, p = 0.000) 2.40 (0.42, 13.63) 4.48
Lung cancer
Harris (1998) 1.21 (0.72, 2.05) 2.17
Minina (2017) 0.92 (0.65, 1.31) 2.92
Saarikoski (1998) 1.02 (0.66, 1.58) 2.54
Sorensen (2004) 0.86 (0.55, 1.34) 2.49
Wang (2003) 1.34 (1.01, 1.79) 3.23
Yang (2004) 1.00 (0.64, 1.57) 2.48
Zienolddiny (2008) 1.98 (1.37, 2.88) 2.83
Subtotal (I-squared = 54.1%, p = 0.042) 1.16 (0.93, 1.45) 18.66
Skin cancer
Ibarrola (2012) 1.34 (0.76, 2.36) 2.02
Lira (2006) 0.52 (0.22, 1.23) 1.22
Marshall (2000) 2.71 (1.29, 5.72) 1.47
Subtotal (I-squared = 75.6%, p = 0.017) 1.27 (0.55, 2.92) 4.71
Pancreatic cancer
Jiao (2007) 0.80 (0.54, 1.18) 2.73
Subtotal (I-squared = .%, p = .) 0.80 (0.54, 1.18) 2.73
Leukaemia
Krajinovic (2002) 0.64 (0.38, 1.07) 2.19
Stanulla (2000) 0.79 (0.36, 1.72) 1.39
Subtotal (I-squared = 0.0%, p = 0.647) 0.68 (0.44, 1.05) 3.58
Head and neck cancer
Li (2007) 1.19 (0.92, 1.53) 3.39
Marciniak (2006) 1.28 (0.63, 2.63) 1.54
Park (1999) 4.23 (0.80, 22.21) 0.41
Park (1999) 0.74 (0.37, 1.52) 1.56
Rajesh (2018) 4.55 (2.33, 8.89) 1.68
Siraj (2008) 1.22 (0.64, 2.31) 1.76
Subtotal (I-squared = 72.6%, p = 0.003) 1.54 (0.94, 2.53) 10.34
Prostate cancer
Oskina (2014) 0.91 (0.64, 1.28) 2.96
Wadelius (1999) 0.95 (0.56, 1.63) 2.12
Subtotal (I-squared = 0.0%, p = 0.880) 0.92 (0.69, 1.23) 5.09
Breast cancer
Van (2008) 1.24 (0.84, 1.83) 2.74
Van (2008) 1.75 (0.46, 6.67) 0.60
Subtotal (I-squared = 0.0%, p = 0.630) 1.27 (0.88, 1.85) 3.35
Overall (I-squared = 67.1%, p = 0.000) 1.17 (1.04, 1.31) 100.00
NOTE: Weights are from random effects analysis
.045
1
22.2

Fig. S7

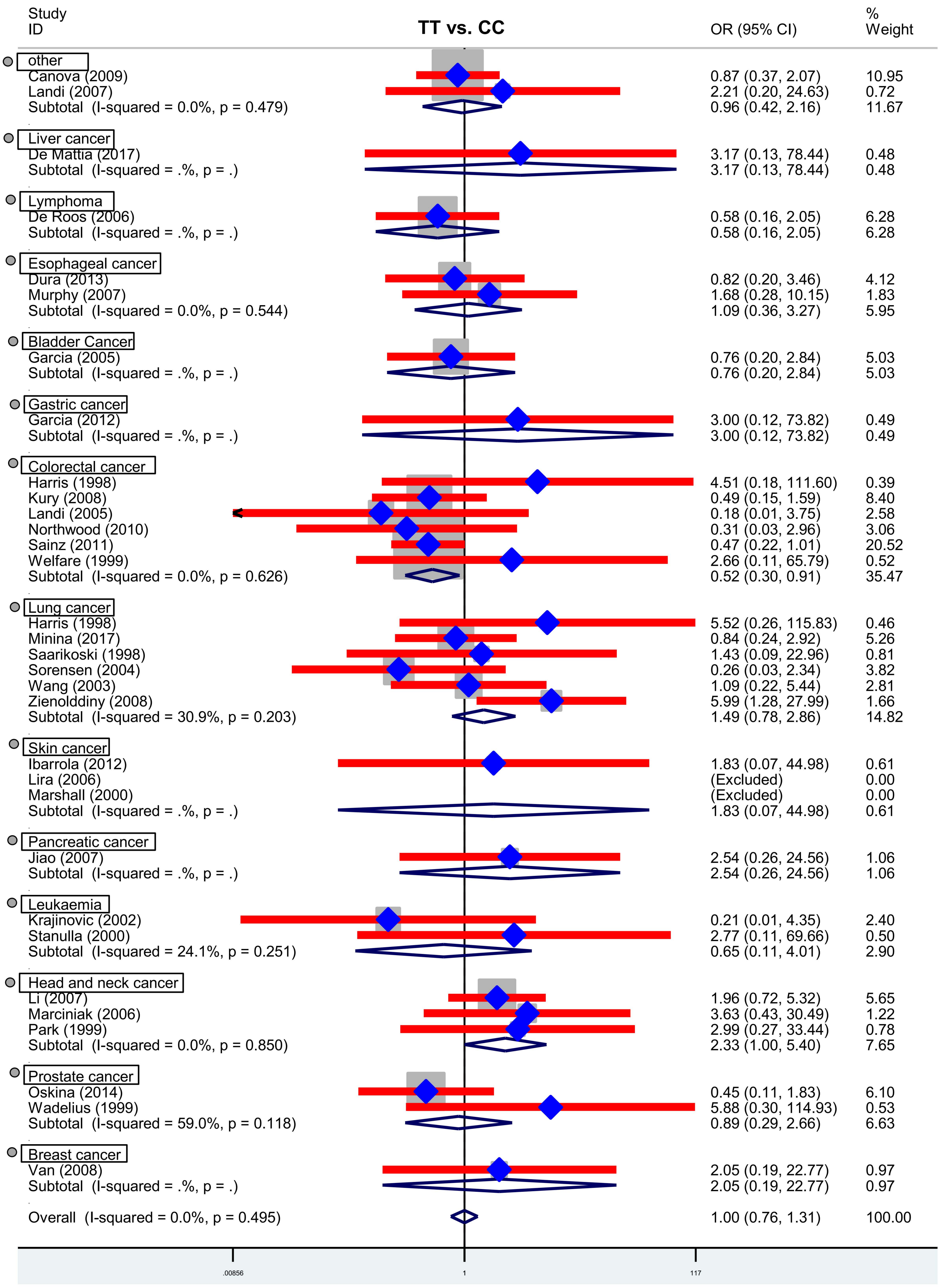
Study
ID
TT vs. CC
OR (95% CI)
%
Weight
other
Canova (2009) 0.87 (0.37, 2.07) 10.95
Landi (2007) 2.21 (0.20, 24.63) 0.72
Subtotal (I-squared = 0.0%, p = 0.479) 0.96 (0.42, 2.16) 11.67
Liver cancer
De Mattia (2017) 3.17 (0.13, 78.44) 0.48
Subtotal (I-squared = .%, p = .) 3.17 (0.13, 78.44) 0.48
Lymphoma
De Roos (2006) 0.58 (0.16, 2.05) 6.28
Subtotal (I-squared = .%, p = .) 0.58 (0.16, 2.05) 6.28
Esophageal cancer
Dura (2013) 0.82 (0.20, 3.46) 4.12
Murphy (2007) 1.68 (0.28, 10.15) 1.83
Subtotal (I-squared = 0.0%, p = 0.544) 1.09 (0.36, 3.27) 5.95
Bladder Cancer
Garcia (2005) 0.76 (0.20, 2.84) 5.03
Subtotal (I-squared = .%, p = .) 0.76 (0.20, 2.84) 5.03
Gastric cancer
Garcia (2012) 3.00 (0.12, 73.82) 0.49
Subtotal (I-squared = .%, p = .) 3.00 (0.12, 73.82) 0.49
Colorectal cancer
Harris (1998) 4.51 (0.18, 111.60) 0.39
Kury (2008) 0.49 (0.15, 1.59) 8.40
Landi (2005) 0.18 (0.01, 3.75) 2.58
Northwood (2010) 0.31 (0.03, 2.96) 3.06
Sainz (2011) 0.47 (0.22, 1.01) 20.52
Welfare (1999) 2.66 (0.11, 65.79) 0.52
Subtotal (I-squared = 0.0%, p = 0.626) 0.52 (0.30, 0.91) 35.47
Lung cancer
Harris (1998) 5.52 (0.26, 115.83) 0.46
Minina (2017) 0.84 (0.24, 2.92) 5.26
Saarikoski (1998) 1.43 (0.09, 22.96) 0.81
Sorensen (2004) 0.26 (0.03, 2.34) 3.82
Wang (2003) 1.09 (0.22, 5.44) 2.81
Zienolddiny (2008) 5.99 (1.28, 27.99) 1.66
Subtotal (I-squared = 30.9%, p = 0.203) 1.49 (0.78, 2.86) 14.82
Skin cancer
Ibarrola (2012) 1.83 (0.07, 44.98) 0.61
Lira (2006) (Excluded) 0.00
Marshall (2000) (Excluded) 0.00
Subtotal (I-squared = .%, p = .) 1.83 (0.07, 44.98) 0.61
Pancreatic cancer
Jiao (2007) 2.54 (0.26, 24.56) 1.06
Subtotal (I-squared = .%, p = .) 2.54 (0.26, 24.56) 1.06
Leukaemia
Krajinovic (2002) 0.21 (0.01, 4.35) 2.40
Stanulla (2000) 2.77 (0.11, 69.66) 0.50
Subtotal (I-squared = 24.1%, p = 0.251) 0.65 (0.11, 4.01) 2.90
Head and neck cancer
Li (2007) 1.96 (0.72, 5.32) 5.65
Marciniak (2006) 3.63 (0.43, 30.49) 1.22
Park (1999) 2.99 (0.27, 33.44) 0.78
Subtotal (I-squared = 0.0%, p = 0.850) 2.33 (1.00, 5.40) 7.65
Prostate cancer
Oskina (2014) 0.45 (0.11, 1.83) 6.10
Wadelius (1999) 5.88 (0.30, 114.93) 0.53
Subtotal (I-squared = 59.0%, p = 0.118) 0.89 (0.29, 2.66) 6.63
Breast cancer
Van (2008) 2.05 (0.19, 22.77) 0.97
Subtotal (I-squared = .%, p = .) 2.05 (0.19, 22.77) 0.97
Overall (I-squared = 0.0%, p = 0.495) 1.00 (0.76, 1.31) 100.00
.00856
1
117

Fig. S8

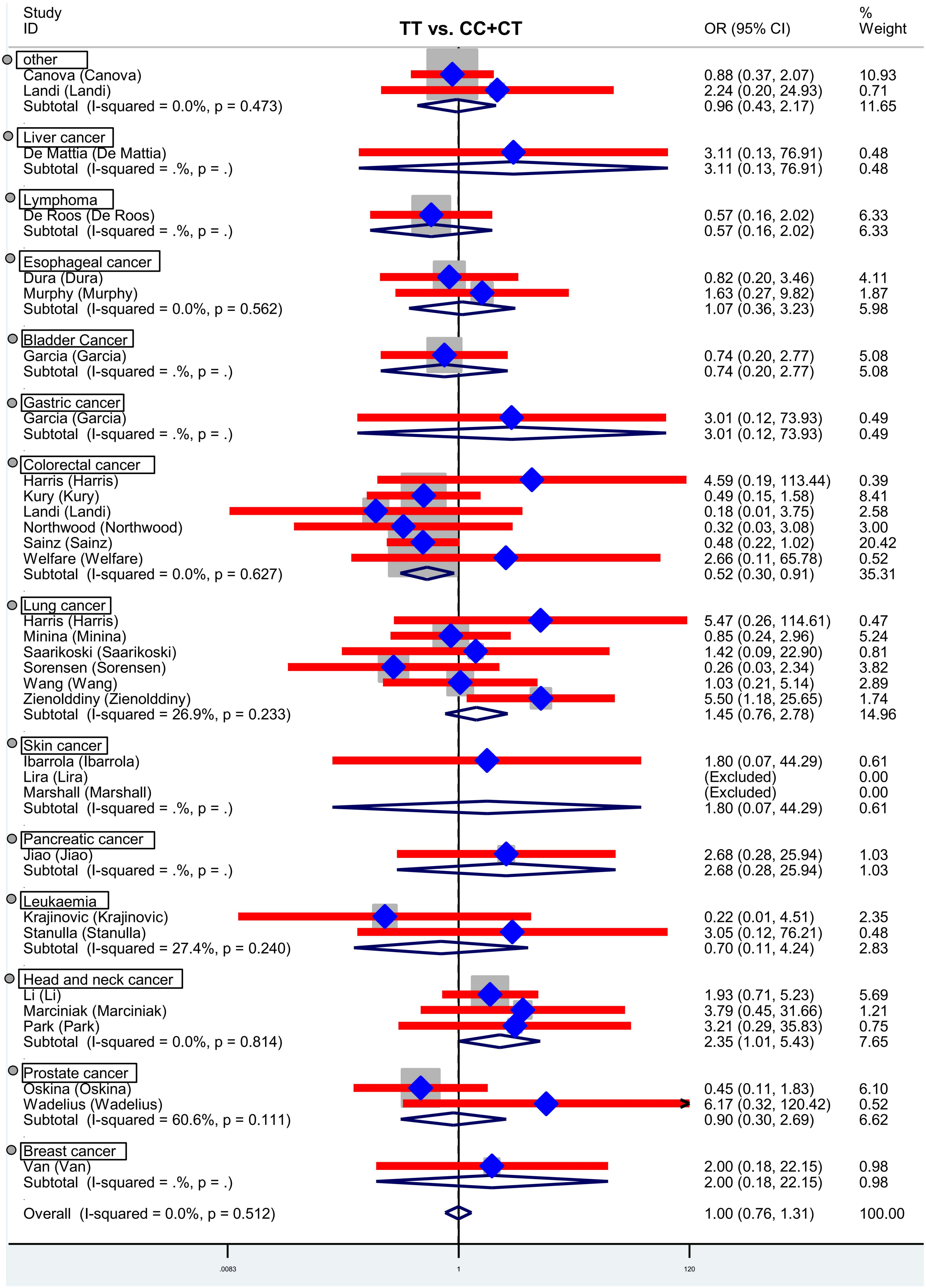
Study
ID
TT vs. CC+CT
OR (95% CI)
%
Weight
other
Canova (Canova) 0.88 (0.37, 2.07) 10.93
Landi (Landi) 2.24 (0.20, 24.93) 0.71
Subtotal (I-squared = 0.0%, p = 0.473) 0.96 (0.43, 2.17) 11.65
Liver cancer
De Mattia (De Mattia) 3.11 (0.13, 76.91) 0.48
Subtotal (I-squared = .%, p = .) 3.11 (0.13, 76.91) 0.48
Lymphoma
De Roos (De Roos) 0.57 (0.16, 2.02) 6.33
Subtotal (I-squared = .%, p = .) 0.57 (0.16, 2.02) 6.33
Esophageal cancer
Dura (Dura) 0.82 (0.20, 3.46) 4.11
Murphy (Murphy) 1.63 (0.27, 9.82) 1.87
Subtotal (I-squared = 0.0%, p = 0.562) 1.07 (0.36, 3.23) 5.98
Bladder Cancer
Garcia (Garcia) 0.74 (0.20, 2.77) 5.08
Subtotal (I-squared = .%, p = .) 0.74 (0.20, 2.77) 5.08
Gastric cancer
Garcia (Garcia) 3.01 (0.12, 73.93) 0.49
Subtotal (I-squared = .%, p = .) 3.01 (0.12, 73.93) 0.49
Colorectal cancer
Harris (Harris) 4.59 (0.19, 113.44) 0.39
Kury (Kury) 0.49 (0.15, 1.58) 8.41
Landi (Landi) 0.18 (0.01, 3.75) 2.58
Northwood (Northwood) 0.32 (0.03, 3.08) 3.00
Sainz (Sainz) 0.48 (0.22, 1.02) 20.42
Welfare (Welfare) 2.66 (0.11, 65.78) 0.52
Subtotal (I-squared = 0.0%, p = 0.627) 0.52 (0.30, 0.91) 35.31
Lung cancer
Harris (Harris) 5.47 (0.26, 114.61) 0.47
Minina (Minina) 0.85 (0.24, 2.96) 5.24
Saarikoski (Saarikoski) 1.42 (0.09, 22.90) 0.81
Sorensen (Sorensen) 0.26 (0.03, 2.34) 3.82
Wang (Wang) 1.03 (0.21, 5.14) 2.89
Zienoldding (Zienoldding) 5.50 (1.18, 25.65) 1.74
Subtotal (I-squared = 26.9%, p = 0.233) 1.45 (0.76, 2.78) 14.96
Skin cancer
Ibarrola (Ibarrola) 1.80 (0.07, 44.29) 0.61
Lira (Lira) (Excluded) 0.00
Marshall (Marshall) (Excluded) 0.00
Subtotal (I-squared = .%, p = .) 1.80 (0.07, 44.29) 0.61
Pancreatic cancer
Jiao (Jiao) 2.68 (0.28, 25.94) 1.03
Subtotal (I-squared = .%, p = .) 2.68 (0.28, 25.94) 1.03
Leukaemia
Krajinovic (Krajinovic) 0.22 (0.01, 4.51) 2.35
Stanulla (Stanulla) 3.05 (0.12, 76.21) 0.48
Subtotal (I-squared = 27.4%, p = 0.240) 0.70 (0.11, 4.24) 2.83
Head and neck cancer
Li (Li) 1.93 (0.71, 5.23) 5.69
Marciniak (Marciniak) 3.79 (0.45, 31.66) 1.21
Park (Park) 3.21 (0.29, 35.83) 0.75
Subtotal (I-squared = 0.0%, p = 0.814) 2.35 (1.01, 5.43) 7.65
Prostate cancer
Oskina (Oskina) 0.45 (0.11, 1.83) 6.10
Wadelius (Wadelius) 6.17 (0.32, 120.42) 0.52
Subtotal (I-squared = 60.6%, p = 0.111) 0.90 (0.30, 2.69) 6.62
Breast cancer
Van (Van) 2.00 (0.18, 22.15) 0.98
Subtotal (I-squared = .%, p = .) 2.00 (0.18, 22.15) 0.98
Overall (I-squared = 0.0%, p = 0.512) 1.00 (0.76, 1.31) 100.00
.0083
1
120

Fig. S9

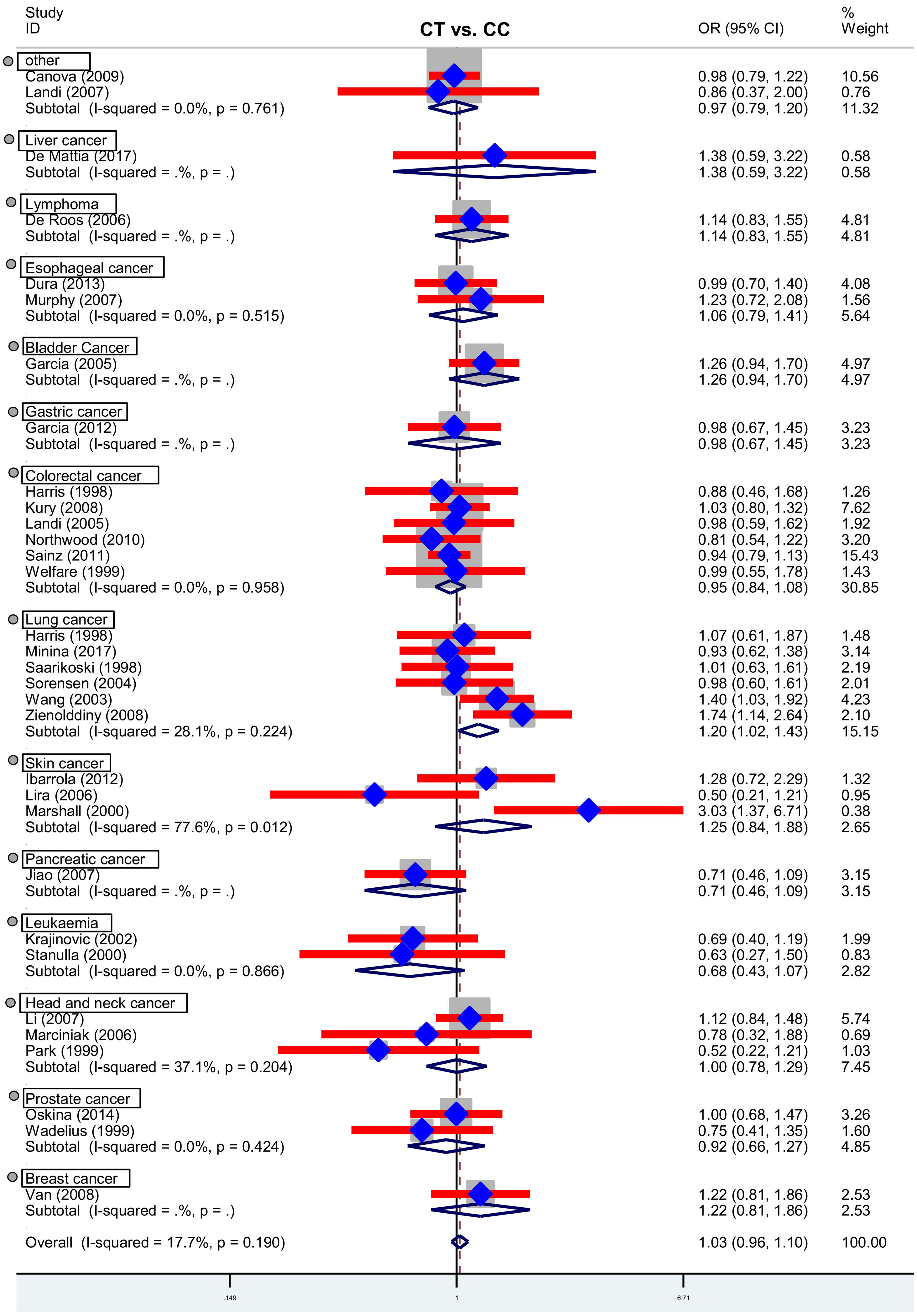
Study ID
CT vs. CC
OR (95% CI)
% Weight
other
Canova (2009) 0.98 (0.79, 1.22) 10.56
Landi (2007) 0.86 (0.37, 2.00) 0.76
Subtotal (I-squared = 0.0%, p = 0.761) 0.97 (0.79, 1.20) 11.32
Liver cancer
De Mattia (2017) 1.38 (0.59, 3.22) 0.58
Subtotal (I-squared = .%, p = .) 1.38 (0.59, 3.22) 0.58
Lymphoma
De Roos (2006) 1.14 (0.83, 1.55) 4.81
Subtotal (I-squared = .%, p = .) 1.14 (0.83, 1.55) 4.81
Esophageal cancer
Dura (2013) 0.99 (0.70, 1.40) 4.08
Murphy (2007) 1.23 (0.72, 2.08) 1.56
Subtotal (I-squared = 0.0%, p = 0.515) 1.06 (0.79, 1.41) 5.64
Bladder Cancer
Garcia (2005) 1.26 (0.94, 1.70) 4.97
Subtotal (I-squared = .%, p = .) 1.26 (0.94, 1.70) 4.97
Gastric cancer
Garcia (2012) 0.98 (0.67, 1.45) 3.23
Subtotal (I-squared = .%, p = .) 0.98 (0.67, 1.45) 3.23
Colorectal cancer
Harris (1998) 0.88 (0.46, 1.68) 1.26
Kury (2008) 1.03 (0.80, 1.32) 7.62
Landi (2005) 0.98 (0.59, 1.62) 1.92
Northwood (2010) 0.81 (0.54, 1.22) 3.20
Sainz (2011) 0.94 (0.79, 1.13) 15.43
Welfare (1999) 0.99 (0.55, 1.78) 1.43
Subtotal (I-squared = 0.0%, p = 0.958) 0.95 (0.84, 1.08) 30.85
Lung cancer
Harris (1998) 1.07 (0.61, 1.87) 1.48
Minina (2017) 0.93 (0.62, 1.38) 3.14
Saarikoski (1998) 1.01 (0.63, 1.61) 2.19
Sorensen (2004) 0.98 (0.60, 1.61) 2.01
Wang (2003) 1.40 (1.03, 1.92) 4.23
Zienolddiny (2008) 1.74 (1.14, 2.64) 2.10
Subtotal (I-squared = 28.1%, p = 0.224) 1.20 (1.02, 1.43) 15.15
Skin cancer
Ibarrola (2012) 1.28 (0.72, 2.29) 1.32
Lira (2006) 0.50 (0.21, 1.21) 0.95
Marshall (2000) 3.03 (1.37, 6.71) 0.38
Subtotal (I-squared = 77.6%, p = 0.012) 1.25 (0.84, 1.88) 2.65
Pancreatic cancer
Jiao (2007) 0.71 (0.46, 1.09) 3.15
Subtotal (I-squared = .%, p = .) 0.71 (0.46, 1.09) 3.15
Leukaemia
Krajinovic (2002) 0.69 (0.40, 1.19) 1.99
Stanulla (2000) 0.63 (0.27, 1.50) 0.83
Subtotal (I-squared = 0.0%, p = 0.866) 0.68 (0.43, 1.07) 2.82
Head and neck cancer
Li (2007) 1.12 (0.84, 1.48) 5.74
Marciniak (2006) 0.78 (0.32, 1.88) 0.69
Park (1999) 0.52 (0.22, 1.21) 1.03
Subtotal (I-squared = 37.1%, p = 0.204) 1.00 (0.78, 1.29) 7.45
Prostate cancer
Oskina (2014) 1.00 (0.68, 1.47) 3.26
Wadelius (1999) 0.75 (0.41, 1.35) 1.60
Subtotal (I-squared = 0.0%, p = 0.424) 0.92 (0.66, 1.27) 4.85
Breast cancer
Van (2008) 1.22 (0.81, 1.86) 2.53
Subtotal (I-squared = .%, p = .) 1.22 (0.81, 1.86) 2.53
Overall (I-squared = 17.7%, p = 0.190) 1.03 (0.96, 1.10) 100.00
.149
1
6.71

Fig. S10

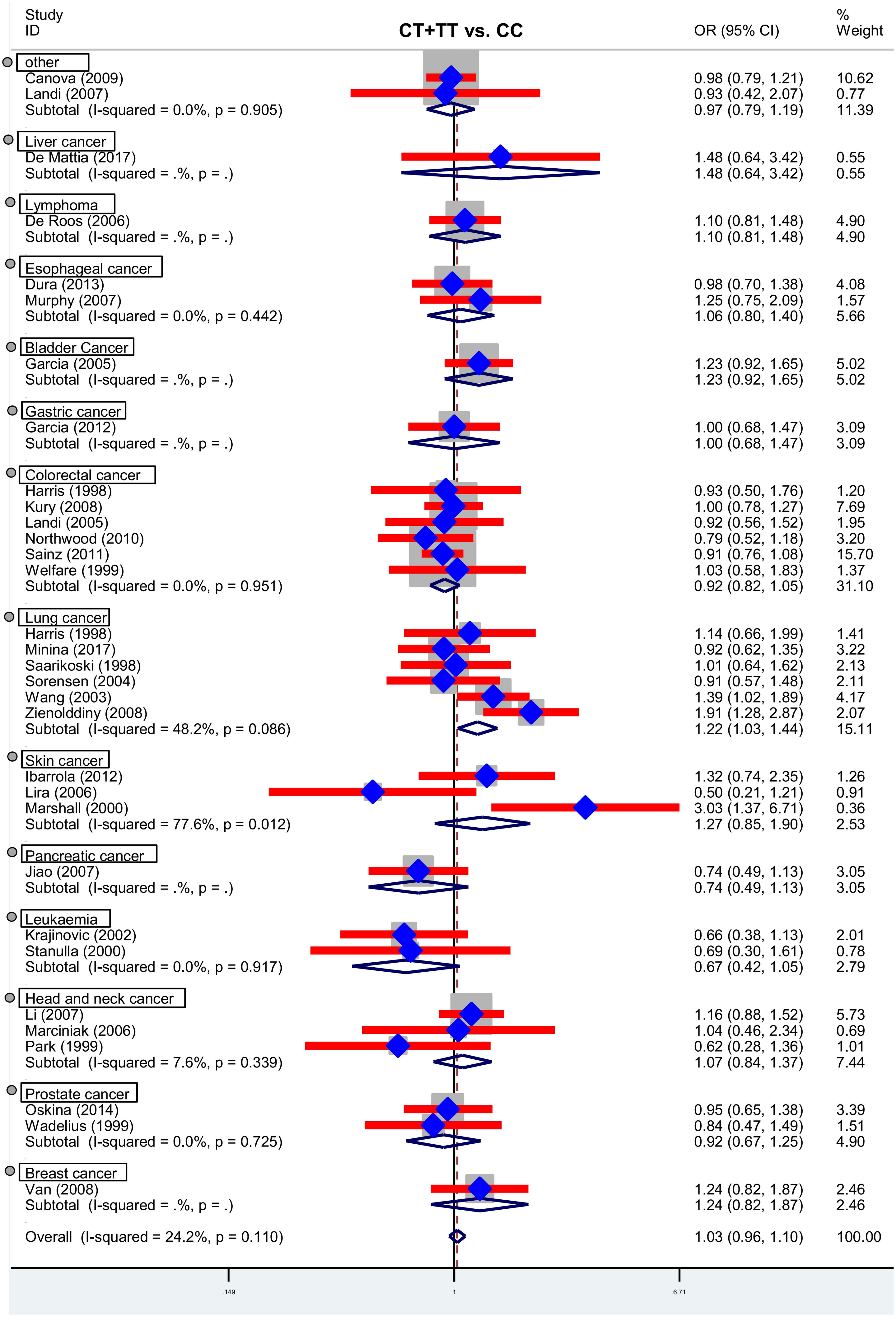
Study
ID
CT+TT vs. CC
OR (95% CI)
%
Weight
other
Canova (2009) 0.98 (0.79, 1.21) 10.62
Landi (2007) 0.93 (0.42, 2.07) 0.77
Subtotal (I-squared = 0.0%, p = 0.905) 0.97 (0.79, 1.19) 11.39
Liver cancer
De Mattia (2017) 1.48 (0.64, 3.42) 0.55
Subtotal (I-squared = .%, p = .) 1.48 (0.64, 3.42) 0.55
Lymphoma
De Roos (2006) 1.10 (0.81, 1.48) 4.90
Subtotal (I-squared = .%, p = .) 1.10 (0.81, 1.48) 4.90
Esophageal cancer
Dura (2013) 0.98 (0.70, 1.38) 4.08
Murphy (2007) 1.25 (0.75, 2.09) 1.57
Subtotal (I-squared = 0.0%, p = 0.442) 1.06 (0.80, 1.40) 5.66
Bladder Cancer
Garcia (2005) 1.23 (0.92, 1.65) 5.02
Subtotal (I-squared = .%, p = .) 1.23 (0.92, 1.65) 5.02
Gastric cancer
Garcia (2012) 1.00 (0.68, 1.47) 3.09
Subtotal (I-squared = .%, p = .) 1.00 (0.68, 1.47) 3.09
Colorectal cancer
Harris (1998) 0.93 (0.50, 1.76) 1.20
Kury (2008) 1.00 (0.78, 1.27) 7.69
Landi (2005) 0.92 (0.56, 1.52) 1.95
Northwood (2010) 0.79 (0.52, 1.18) 3.20
Sainz (2011) 0.91 (0.76, 1.08) 15.70
Welfare (1999) 1.03 (0.58, 1.83) 1.37
Subtotal (I-squared = 0.0%, p = 0.951) 0.92 (0.82, 1.05) 31.10
Lung cancer
Harris (1998) 1.14 (0.66, 1.99) 1.41
Minina (2017) 0.92 (0.62, 1.35) 3.22
Saarikoski (1998) 1.01 (0.64, 1.62) 2.13
Sorensen (2004) 0.91 (0.57, 1.48) 2.11
Wang (2003) 1.39 (1.02, 1.89) 4.17
Zienolddiny (2008) 1.91 (1.28, 2.87) 2.07
Subtotal (I-squared = 48.2%, p = 0.086) 1.22 (1.03, 1.44) 15.11
Skin cancer
Ibarrola (2012) 1.32 (0.74, 2.35) 1.26
Lira (2006) 0.50 (0.21, 1.21) 0.91
Marshall (2000) 3.03 (1.37, 6.71) 0.36
Subtotal (I-squared = 77.6%, p = 0.012) 1.27 (0.85, 1.90) 2.53
Pancreatic cancer
Jiao (2007) 0.74 (0.49, 1.13) 3.05
Subtotal (I-squared = .%, p = .) 0.74 (0.49, 1.13) 3.05
Leukaemia
Krajinovic (2002) 0.66 (0.38, 1.13) 2.01
Stanulla (2000) 0.69 (0.30, 1.61) 0.78
Subtotal (I-squared = 0.0%, p = 0.917) 0.67 (0.42, 1.05) 2.79
Head and neck cancer
Li (2007) 1.16 (0.88, 1.52) 5.73
Marciniak (2006) 1.04 (0.46, 2.34) 0.69
Park (1999) 0.62 (0.28, 1.36) 1.01
Subtotal (I-squared = 7.6%, p = 0.339) 1.07 (0.84, 1.37) 7.44
Prostate cancer
Oskina (2014) 0.95 (0.65, 1.38) 3.39
Wadelius (1999) 0.84 (0.47, 1.49) 1.51
Subtotal (I-squared = 0.0%, p = 0.725) 0.92 (0.67, 1.25) 4.90
Breast cancer
Van (2008) 1.24 (0.82, 1.87) 2.46
Subtotal (I-squared = .%, p = .) 1.24 (0.82, 1.87) 2.46
Overall (I-squared = 24.2%, p = 0.110) 1.03 (0.96, 1.10) 100.00
.149
1
6.71

Fig. S11

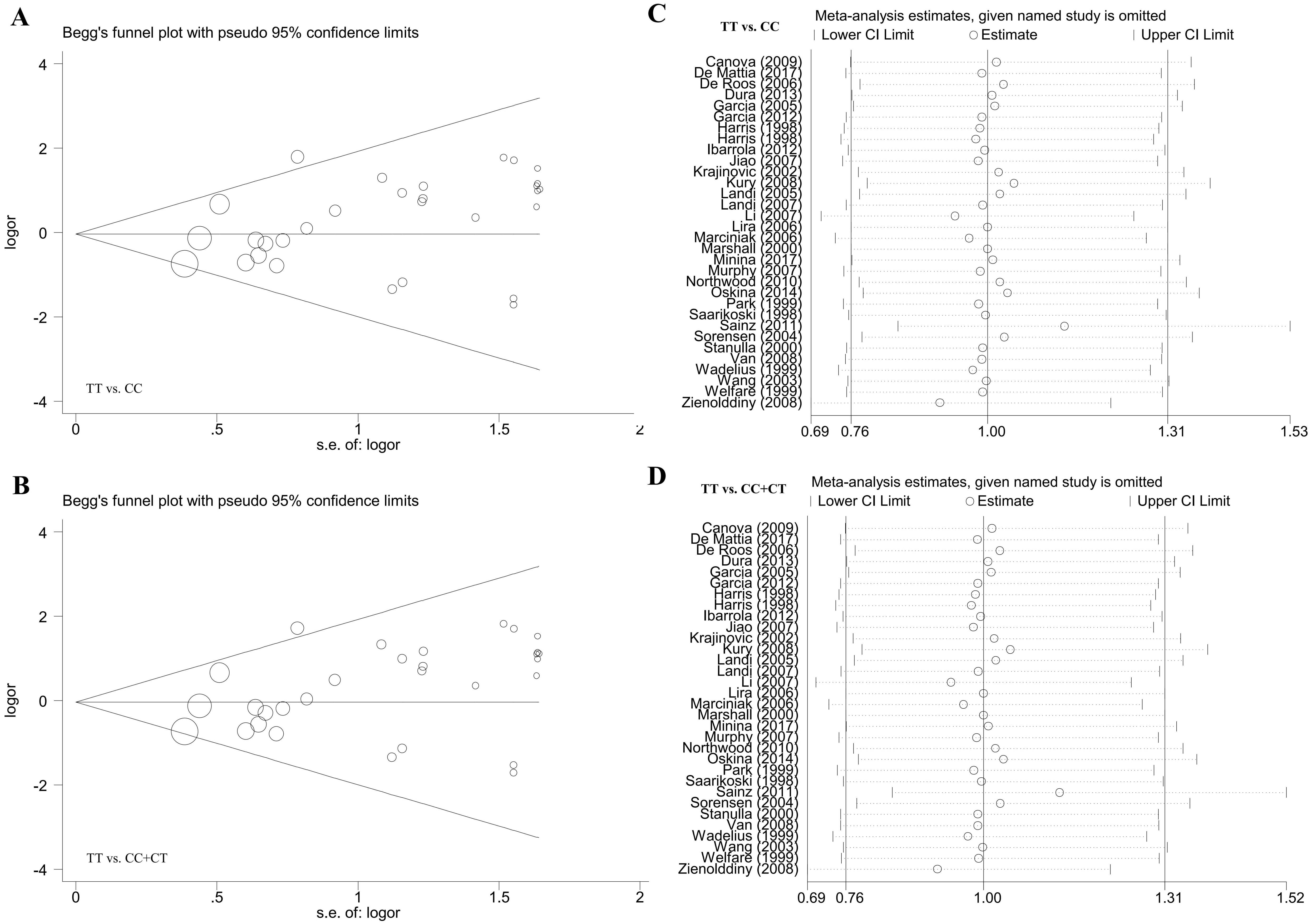
A
Begg's funnel plot with pseudo 95% confidence limits
logor
s.e. of: logor
TT vs. CC
B
Begg's funnel plot with pseudo 95% confidence limits
logor
s.e. of: logor
TT vs. CC+CT
C
TT vs. CC
Meta-analysis estimates, given named study is omitted
Lower CI Limit
Estimate
Upper CI Limit
Canova (2009)
De Mattia (2017)
De Roos (2006)
Dura (2013)
Garcia (2005)
Garcia (2012)
Harris (1998)
Harris (1998)
Ibarrola (2012)
Jiao (2007)
Krajinovic (2002)
Kury (2008)
Landi (2005)
Landi (2007)
Li (2007)
Lira (2006)
Marciniak (2006)
Marshall (2000)
Minina (2017)
Murphy (2007)
Northwood (2010)
Oskina (2014)
Park (1999)
Saarikoski (1998)
Sainz (2011)
Sorensen (2004)
Stanulla (2000)
Van (2008)
Wadelius (1999)
Wang (2003)
Welfare (1999)
Zienolddiny (2008)
0.69 0.76 1.00 1.31 1.53
D
TT vs. CC+CT
Meta-analysis estimates, given named study is omitted
Lower CI Limit
Estimate
Upper CI Limit
Canova (2009)
De Mattia (2017)
De Roos (2006)
Dura (2013)
Garcia (2005)
Garcia (2012)
Harris (1998)
Harris (1998)
Ibarrola (2012)
Jiao (2007)
Krajinovic (2002)
Kury (2008)
Landi (2005)
Landi (2007)
Li (2007)
Lira (2006)
Marciniak (2006)
Marshall (2000)
Minina (2017)
Murphy (2007)
Northwood (2010)
Oskina (2014)
Park (1999)
Saarikoski (1998)
Sainz (2011)
Sorensen (2004)
Stanulla (2000)
Van (2008)
Wadelius (1999)
Wang (2003)
Welfare (1999)
Zienolddiny (2008)
0.69 0.76 1.00 1.31 1.52

Fig. S12

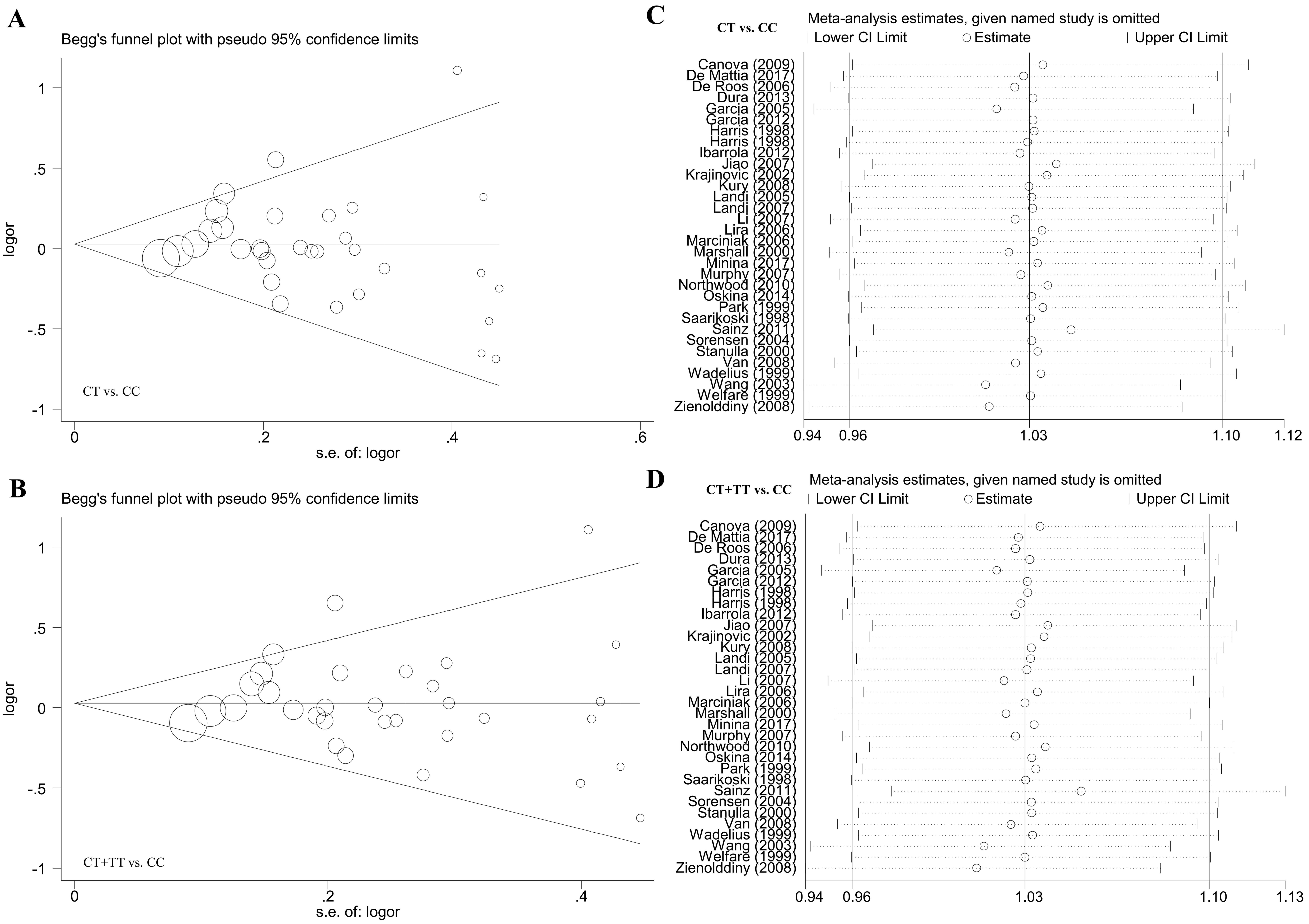

A
Begg's funnel plot with pseudo 95% confidence limits
logor
s.e. of: logor
CT vs. CC
B
Begg's funnel plot with pseudo 95% confidence limits
logor
s.e. of: logor
CT+TT vs. CC
C
CT vs. CC
Meta-analysis estimates, given named study is omitted
Lower CI Limit
Estimate
Upper CI Limit
Canova (2009)
De Mattia (2017)
De Roos (2006)
Dura (2013)
Garcia (2005)
Garcia (2012)
Harris (1998)
Harris (1998)
Ibarrola (2012)
Jiao (2007)
Krajinovic (2002)
Kury (2008)
Landi (2005)
Landi (2007)
Li (2007)
Lira (2006)
Marciniak (2006)
Marshall (2000)
Minina (2017)
Murphy (2007)
Northwood (2010)
Oskina (2014)
Park (1999)
Saarikoski (1998)
Sainz (2011)
Sorensen (2004)
Stanulla (2000)
Van (2008)
Wadelius (1999)
Wang (2003)
Welfare (1999)
Zienolddiny (2008)
0.94 0.96 1.03 1.10 1.12
D
CT+TT vs. CC
Meta-analysis estimates, given named study is omitted
Lower CI Limit
Estimate
Upper CI Limit
Canova (2009)
De Mattia (2017)
De Roos (2006)
Dura (2013)
Garcia (2005)
Garcia (2012)
Harris (1998)
Harris (1998)
Ibarrola (2012)
Jiao (2007)
Krajinovic (2002)
Kury (2008)
Landi (2005)
Landi (2007)
Li (2007)
Lira (2006)
Marciniak (2006)
Marshall (2000)
Minina (2017)
Murphy (2007)
Northwood (2010)
Oskina (2014)
Park (1999)
Saarikoski (1998)
Sainz (2011)
Sorensen (2004)
Stanulla (2000)
Van (2008)
Wadelius (1999)
Wang (2003)
Welfare (1999)
Zienolddiny (2008)
0.94 0.96 1.03 1.10 1.13
