# Supplementary material for: Comprehensive Analysis of the Association Between the rs1138272 Polymorphism of the GSTP1 Gene and Cancer Susceptibility
Source: Front Physiol. 2019 Jan 25;9:1897. doi: 10.3389/fphys.2018.01897 (PMC6355699; doi:10.3389/fphys.2018.01897)
Supplement: TABLE S1 — Database searching terms (up to September 2018). [file Data_Sheet_2.docx]

Table S1. Database searching terms (up to Sep 2018).

| 1. **PubMed🡪[736 records]**   Search ((((((((((((((((((((Neoplasms[MeSH Major Topic]) OR Neoplasia) OR Neoplasias) OR Neoplasm) OR Tumors) OR Tumor) OR Cancer) OR Cancers) OR Malignant Neoplasms) OR Malignant Neoplasm) OR Neoplasm, Malignant) OR Neoplasms, Malignant) OR Malignancy) OR Malignancies) OR Benign Neoplasms) OR Neoplasms, Benign) OR Benign Neoplasm) OR Neoplasm, Benign)) AND (((((((((((((Glutathione S-Transferase pi[MeSH Major Topic]) OR Glutathione S Transferase pi) OR GST Class-phi) OR Class-phi, GST) OR GST Class phi) OR Glutathione Transferase P1-1) OR Glutathione Transferase P1 1) OR Transferase P1-1, Glutathione) OR GSTP1 Glutathione D-Transferase) OR D-Transferase, GSTP1 Glutathione) OR GSTP1 Glutathione D Transferase) OR Glutathione D-Transferase, GSTP1) OR GSTP1)) AND (((((((((((((((((((Polymorphism, Single Nucleotide) OR Nucleotide Polymorphism, Single) OR Nucleotide Polymorphisms, Single) OR Polymorphisms, Single Nucleotide) OR Single Nucleotide Polymorphisms) OR SNPs) OR SNP) OR Single Nucleotide Polymorphism) OR Mutation) OR variant) OR rs1138272) OR p.Ala114Val) OR Alanine114valine) OR Ala114Val) OR A114V) OR c.341C>T) OR C341T) OR C7514T) OR 7514C>T) |
| --- |
| 1. **Embase🡪 [484 records]**   #1 'malignant neoplasm':ti,ab,kw OR 'cancer':ti,ab,kw OR 'cancers':ti,ab,kw OR 'malignant neoplasia':ti,ab,kw OR 'malignant neoplastic disease':ti,ab,kw OR 'malignant tumor':ti,ab,kw OR 'malignant tumour':ti,ab,kw OR 'neoplasia, malignant':ti,ab,kw OR 'tumor, malignant':ti,ab,kw OR 'tumour, malignant':ti,ab,kw [4,537,757]  #2 'glutathione transferase p1':ti,ab,kw OR 'glutathione s transferase p 1':ti,ab,kw OR 'glutathione s transferase p1':ti,ab,kw OR 'glutathione s transferase pi':ti,ab,kw OR 'glutathione s-transferase pi':ti,ab,kw OR 'glutathione transferase p 1':ti,ab,kw OR 'glutathione transferase pi':ti,ab,kw OR 'gst p 1':ti,ab,kw OR 'alanine114valine':ti,ab,kw OR 'gst p1':ti,ab,kw OR 'gstp1':ti,ab,kw [4,818]  #3 ('single nucleotide polymorphism':ti,ab,kw OR 'polymorphism, single nucleotide':ti,ab,kw OR 'snp':ti,ab,kw OR 'snps':ti,ab,kw OR 'mutation':ti,ab,kw OR 'variant':ti,ab,kw OR 'rs1138272':ti,ab,kw OR 'alanine114valine':ti,ab,kw OR 'ala114val':ti,ab,kw OR 'a114v':ti,ab,kw OR 'c341t':ti,ab,kw) [714,965]  #4 #1 AND #2 AND #3 [484] |
| 1. **Cochrane🡪 [60 records]**   #1 (“Neoplasms”) OR (“Cancer”) OR (“Cancers”) (Word variations have been searched) [143,880]  #2 ("Glutathione S-Transferase pi") OR ("Glutathione S Transferase pi") OR ("Glutathione Transferase P1-1") OR ("Glutathione Transferase P1 1") OR ("GSTP1") (Word variations have been searched) [103]  #3 #1 and #2 [60] |
| 1. **Scopus🡪 [723 records]**   (TITLE-ABS-KEY ("Neoplasms" OR "Cancer" OR "Cancers") AND TITLE-ABS-KEY("Glutathione S-Transferase pi" OR "Glutathione S Transferase pi" OR "Glutathione Transferase P1-1" OR "Glutathione Transferase P1 1" OR "GSTP1") AND TITLE-ABS-KEY ("Single Nucleotide Polymorphisms" OR "SNPs" OR "SNP" OR "Single Nucleotide Polymorphism" OR "Mutation" OR "variant" OR "rs1138272")) AND DOCTYPE (ar) |
| 1. **WOS🡪 [801 records]**   ((TOPIC: (("Neoplasms" OR "Cancer") OR "Cancers") AND TOPIC: (((("Glutathione S-Transferase pi" OR "Glutathione S Transferase pi") OR "Glutathione Transferase P1-1") OR "Glutathione Transferase P1 1") OR "GSTP1")) AND TOPIC: (((((("Single Nucleotide Polymorphisms" OR "SNPs") OR "SNP") OR "Single Nucleotide Polymorphism") OR "Mutation") OR "variant") OR "rs11038172"))  Refined by: DOCUMENT TYPES: (ARTICLE)  Timespan: All years. Databases: WOS, KJD, RSCI, SCIELO.  Search language=Auto |

**Note:** WOS, web of science.

Table S2. Subgroup analysis by control source in the overall population.

| **Subgroup** | **Models** | **Study(N)** | **Case(N)** | **Control(N)** | **I^2^** | ***P*-heterogeneity** | **OR [95% CI]** | ***P*-association** |
| --- | --- | --- | --- | --- | --- | --- | --- | --- |
| **PB** | allele T vs. allele C | 29 | 11,074 | 12,285 | 64.3% | <0.001 | 1.12 [0.99-1.26] | 0.068 |
|  | carrier T vs. carrier C | 29 | 11,074 | 12,285 | 38.6% | 0.019 | 1.06 [0.96-1.17] | 0.215 |
|  | TT vs. CC | 29 | 11,074 | 12,285 | 38.1% | 0.021 | 1.45 [1.11-1.89] | 0.006 |
|  | TT vs. CC+CT | 29 | 11,074 | 12,285 | 36.4% | 0.028 | 1.44 [1.10-1.87] | 0.007 |
|  | CT vs. CC | 29 | 11,074 | 12,285 | 31.6% | 0.054 | 1.03 [0.94-1.14] | 0.519 |
|  | CT+TT vs. CC | 29 | 11,074 | 12,285 | 52.0% | 0.001 | 1.08 [0.96-1.20] | 0.202 |
| **HB** | allele T vs. allele C | 12 | 3,025 | 3,018 | 72.4% | <0.001 | 1.40 [1.00-1.95] | 0.050 |
|  | carrier T vs. carrier C | 12 | 3,025 | 3,018 | 61.1% | 0.003 | 1.32 [0.98-1.78] | 0.065 |
|  | TT vs. CC | 9 | 2,816 | 2,640 | 0.0% | 0.489 | 1.88 [0.96-3.67] | 0.066 |
|  | TT vs. CC+CT | 9 | 2,816 | 2,640 | 0.0% | 0.608 | 1.74 [0.88-3.41] | 0.109 |
|  | CT vs. CC | 12 | 3,025 | 3,018 | 71.2% | <0.001 | 1.36 [0.95-1.93] | 0.093 |
|  | CT+TT vs. CC | 12 | 3,025 | 3,018 | 73.3% | <0.001 | 1.40 [0.98-2.00] | 0.067 |

**Note:** PB, population-based control; HB, hospital-based control; N, number; *P*-heterogeneity, *P* value of heterogeneity test.

OR, odds ratio; CI, confidence interval; *P*-association, *P* value of association test.

Table S3. Subgroup analysis by cancer type in the overall population.

| **Subgroup** | **Models** | **Study(N)** | **Case(N)** | **Control(N)** | **I^2^** | ***P*-heterogeneity** | **OR [95% CI]** | ***P*-association** |
| --- | --- | --- | --- | --- | --- | --- | --- | --- |
| **Colorectal cancer** | allele T vs. allele C | 9 | 4,858 | 4,998 | 32.8% | 0.155 | 0.98 [0.85-1.14] | 0.817 |
|  | carrier T vs. carrier C | 9 | 4,858 | 4,998 | 0.0% | 0.466 | 0.97 [0.87-1.08] | 0.569 |
|  | TT vs. CC | 9 | 4,858 | 4,998 | 10.9% | 0.344 | 0.71 [0.44-1.14] | 0.158 |
|  | TT vs. CC+CT | 9 | 4,858 | 4,998 | 7.7% | 0.371 | 0.71 [0.44-1.14] | 0.159 |
|  | CT vs. CC | 9 | 4,858 | 4,998 | 7.1% | 0.376 | 0.98 [0.87-1.11] | 0.807 |
|  | CT+TT vs. CC | 9 | 4,858 | 4,998 | 22.6% | 0.242 | 0.98 [0.86-1.13] | 0.812 |
| **Lung cancer** | allele T vs. allele C | 7 | 2,123 | 2,266 | 54.1% | 0.042 | 1.16 [0.93-1.45] | 0.186 |
|  | carrier T vs. carrier C | 7 | 2,123 | 2,266 | 26.8% | 0.224 | 1.14 [0.95-1.38] | 0.163 |
|  | TT vs. CC | 7 | 2,123 | 2,266 | 21.2% | 0.268 | 1.60 [0.88-2.93] | 0.125 |
|  | TT vs. CC+CT | 7 | 2,123 | 2,266 | 17.7% | 0.295 | 1.57 [0.86-2.88] | 0.141 |
|  | CT vs. CC | 7 | 2,123 | 2,266 | 31.8% | 0.185 | 1.14 [0.93-1.39] | 0.207 |
|  | CT+TT vs. CC | 7 | 2,123 | 2,266 | 44.7% | 0.093 | 1.16 [0.93-1.44] | 0.193 |
| **Head and neck cancer** | allele T vs. allele C | 6 | 1,190 | 1,827 | 72.6% | 0.003 | 1.54 [0.94-2.53] | 0.088 |
|  | carrier T vs. carrier C | 6 | 1,190 | 1,827 | 61.6% | 0.023 | 1.34 [0.85-2.13] | 0.209 |
|  | TT vs. CC | 6 | 1,190 | 1,827 | 0.0% | 0.773 | 3.11 [1.57-6.19] | 0.001 |
|  | TT vs. CC+CT | 6 | 1,190 | 1,827 | 0.0% | 0.818 | 3.07 [1.54-6.11] | 0.001 |
|  | CT vs. CC | 6 | 1,190 | 1,827 | 64.1% | 0.016 | 1.70 [0.70-1.96] | 0.548 |
|  | CT+TT vs. CC | 6 | 1,190 | 1,827 | 69.2% | 0.006 | 1.38 [0.82-2.32] | 0.229 |

**Note:** N, number; *P*-heterogeneity, *P* value of heterogeneity test; OR, odds ratio; CI, confidence interval; *P*-association, *P* value of association test.

Table S4. Subgroup analysis by control source in the Caucasian population.

| **Subgroup** | **Models** | **Study(N)** | **Case(N)** | **Control(N)** | **I^2^** | ***P*-heterogeneity** | **OR [95% CI]** | ***P*-association** |
| --- | --- | --- | --- | --- | --- | --- | --- | --- |
| **PB** | allele T vs. allele C | 22 | 9,488 | 9,771 | 32.2% | 0.074 | 1.01 [0.94-1.09] | 0.708 |
|  | carrier T vs. carrier C | 22 | 9,488 | 9,771 | 0.0% | 0.564 | 1.01 [0.94-1.10] | 0.720 |
|  | TT vs. CC | 22 | 9,488 | 9,771 | 15.6% | 0.253 | 1.00 [0.73-1.36] | 0.981 |
|  | TT vs. CC+CT | 22 | 9,488 | 9,771 | 13.8% | 0.276 | 0.99 [0.73-1.36] | 0.969 |
|  | CT vs. CC | 22 | 9,488 | 9,771 | 0.0% | 0.472 | 1.02 [0.94-1.10] | 0.666 |
|  | CT+TT vs. CC | 22 | 9,488 | 9,771 | 16.7% | 0.239 | 1.02 [0.94-1.10] | 0.679 |
| **HB** | allele T vs. allele C | 8 | 2,706 | 2,580 | 43.8% | 0.087 | 1.11 [0.94-1.32] | 0.211 |
|  | carrier T vs. carrier C | 8 | 2,706 | 2,580 | 33.4% | 0.162 | 1.11 [0.93-1.33] | 0.248 |
|  | TT vs. CC | 6 | 2,551 | 2,276 | 0.0% | 0.698 | 1.06 [0.46-2.45] | 0.891 |
|  | TT vs. CC+CT | 6 | 2,551 | 2,276 | 0.0% | 0.672 | 1.06 [0.46-2.46] | 0.883 |
|  | CT vs. CC | 6 | 2,551 | 2,276 | 54.4% | 0.032 | 1.12 [0.94-1.35] | 0.211 |
|  | CT+TT vs. CC | 6 | 2,551 | 2,276 | 50.7% | 0.048 | 1.12 [0.94-1.34] | 0.206 |

**Note:** PB, population-based control; HB, hospital-based control; N, number; *P*-heterogeneity, *P* value of heterogeneity test.

OR, odds ratio; CI, confidence interval; *P*-association, *P* value of association test.

Table S5. Subgroup analysis by cancer type in the Caucasian population.

| **Subgroup** | **Models** | **Study(N)** | **Case(N)** | **Control(N)** | **I^2^** | ***P*-heterogeneity** | **OR [95% CI]** | ***P*-association** |
| --- | --- | --- | --- | --- | --- | --- | --- | --- |
| **Colorectal cancer** | allele T vs. allele C | 6 | 3,783 | 3,898 | 0.0% | 0.924 | 0.91 [0.81-1.02] | 0.095 |
|  | carrier T vs. carrier C | 6 | 3,783 | 3,898 | 0.0% | 0.976 | 0.93 [0.82-1.05] | 0.266 |
|  | TT vs. CC | 6 | 3,783 | 3,898 | 0.0% | 0.626 | 0.52 [0.30-0.91] | 0.021 |
|  | TT vs. CC+CT | 6 | 3,783 | 3,898 | 0.0% | 0.627 | 0.52 [0.30-0.91] | 0.023 |
|  | CT vs. CC | 6 | 3,783 | 3,898 | 0.0% | 0.958 | 0.95 [0.84-1.08] | 0.435 |
|  | CT+TT vs. CC | 6 | 3,783 | 3,898 | 0.0% | 0.951 | 0.92 [0.82-1.05] | 0.218 |
| **Lung cancer** | allele T vs. allele C | 6 | 1,894 | 2,037 | 59.8% | 0.029 | 1.21 [1.04-1.42] | 0.015 |
|  | carrier T vs. carrier C | 6 | 1,894 | 2,037 | 32.4% | 0.193 | 1.18 [1.00-1.40] | 0.044 |
|  | TT vs. CC | 6 | 1,894 | 2,037 | 30.9% | 0.203 | 1.49 [0.78-2.86] | 0.229 |
|  | TT vs. CC+CT | 6 | 1,894 | 2,037 | 26.9% | 0.233 | 1.45 [0.76-2.78] | 0.264 |
|  | CT vs. CC | 6 | 1,894 | 2,037 | 28.1% | 0.224 | 1.20 [1.02-1.43] | 0.032 |
|  | CT+TT vs. CC | 6 | 1,894 | 2,037 | 48.2% | 0.086 | 1.22 [1.03-1.44] | 0.020 |
| **Head and neck cancer** | allele T vs. allele C | 3 | 1,009 | 1,054 | 0.0% | 0.453 | 1.14 [0.91-1.43] | 0.256 |
|  | carrier T vs. carrier C | 3 | 1,009 | 1,054 | 0.0% | 0.444 | 1.07 [0.84-1.37] | 0.560 |
|  | TT vs. CC | 3 | 1,009 | 1,054 | 0.0% | 0.850 | 2.33 [1.00-5.40] | 0.049 |
|  | TT vs. CC+CT | 3 | 1,009 | 1,054 | 0.0% | 0.814 | 2.35 [1.01-5.43] | 0.047 |
|  | CT vs. CC | 3 | 1,009 | 1,054 | 37.1% | 0.204 | 1.00 [0.78-1.29] | 0.987 |
|  | CT+TT vs. CC | 3 | 1,009 | 1,054 | 7.6% | 0.339 | 1.07 [0.84-1.37] | 0.564 |

**Note:** N, number; *P*-heterogeneity, *P* value of heterogeneity test; OR, odds ratio; CI, confidence interval; *P*-association, *P* value of association test.

Table S6. Subgroup analysis by cancer type using the Caucasian cases and population-based negative controls.

| **Subgroup** | **Models** | **Study(N)** | **Case(N)** | **Control(N)** | **I^2^** | ***P*-heterogeneity** | **OR [95% CI]** | ***P*-association** |
| --- | --- | --- | --- | --- | --- | --- | --- | --- |
| **Colorectal cancer** | allele T vs. allele C | 5 | 3,423 | 3,573 | 0.0% | 0.847 | 0.91 [0.80-1.02] | 0.114 |
|  | carrier T vs. carrier C | 5 | 3,423 | 3,573 | 0.0% | 0.936 | 0.93 [0.82-1.06] | 0.285 |
|  | TT vs. CC | 5 | 3,423 | 3,573 | 0.0% | 0.555 | 0.54 [0.31-0.96] | 0.037 |
|  | TT vs. CC+CT | 5 | 3,423 | 3,573 | 0.0% | 0.557 | 0.55 [0.31-0.97] | 0.040 |
|  | CT vs. CC | 5 | 3,423 | 3,573 | 0.0% | 0.903 | 0.95 [0.83-1.08] | 0.432 |
|  | CT+TT vs. CC | 5 | 3,423 | 3,573 | 0.0% | 0.888 | 0.92 [0.82-1.05] | 0.234 |
| **Lung cancer** | allele T vs. allele C | 6 | 1,894 | 2,037 | 59.8% | 0.029 | 1.21 [1.04-1.42] | 0.015 |
|  | carrier T vs. carrier C | 6 | 1,894 | 2,037 | 32.4% | 0.193 | 1.18 [1.00-1.40] | 0.044 |
|  | TT vs. CC | 6 | 1,894 | 2,037 | 30.9% | 0.203 | 1.49 [0.78-2.86] | 0.229 |
|  | TT vs. CC+CT | 6 | 1,894 | 2,037 | 26.9% | 0.233 | 1.45 [0.76-2.78] | 0.264 |
|  | CT vs. CC | 6 | 1,894 | 2,037 | 28.1% | 0.224 | 1.20 [1.02-1.43] | 0.032 |
|  | CT+TT vs. CC | 6 | 1,894 | 2,037 | 48.2% | 0.086 | 1.22 [1.03-1.44] | 0.020 |

**Note:** N, number; *P*-heterogeneity, *P* value of heterogeneity test; OR, odds ratio; CI, confidence interval; *P*-association, *P* value of association test.

Table S7. Publication bias analysis in the overall population.

| **Models** | **Study(N)** | **z-Begg's Test** | ***P*-Begg's Test** | **t-Egger's test** | ***P*-Egger's test** |
| --- | --- | --- | --- | --- | --- |
| allele T vs. allele C | 43 | 2.20 | 0.028 | 2.60 | 0.013 |
| carrier T vs. carrier C | 43 | 1.76 | 0.079 | 2.18 | 0.035 |
| TT vs. CC | 40 | 1.74 | 0.083 | 2.62 | 0.013 |
| TT vs. CC+CT | 40 | 1.60 | 0.110 | 2.61 | 0.013 |
| CT vs. CC | 43 | 0.50 | 0.615 | 1.30 | 0.202 |
| CT+TT vs. CC | 43 | 1.65 | 0.098 | 2.06 | 0.046 |

**Note:** N, number; z-Begg's Test, continuity corrected z value of Begg's Test; *P*-Begg's Test, continuity corrected

*P* value of Begg's Test; t-Egger's test, t value of Egger's Test; *P*- Egger's Test, *P* value of Egger's Test.

Table S8. Publication bias analysis in the Caucasian population.

| **Models** | **Study(N)** | **z-Begg's Test** | ***P*-Begg's Test** | **t-Egger's test** | ***P*-Egger's test** |
| --- | --- | --- | --- | --- | --- |
| allele T vs. allele C | 32 | 0.02 | 0.987 | 0.64 | 0.524 |
| carrier T vs. carrier C | 32 | 0.21 | 0.833 | 0.13 | 0.896 |
| TT vs. CC | 30 | 2.11 | 0.035 | 2.38 | 0.024 |
| TT vs. CC+CT | 30 | 2.07 | 0.038 | 2.44 | 0.021 |
| CT vs. CC | 32 | 1.28 | 0.200 | -0.62 | 0.539 |
| CT+TT vs. CC | 32 | 0.47 | 0.638 | 0.03 | 0.973 |

**Note:** N, number; z-Begg's Test, continuity corrected z value of Begg's Test; *P*-Begg's Test, continuity corrected

*P* value of Begg's Test; t-Egger's test, t value of Egger's Test; *P*- Egger's Test, *P* value of Egger's Test.

Table S9. Publication bias analysis in the Caucasian cases and population-based negative controls.

| **Models** | **Study(N)** | **z-Begg's Test** | ***P*-Begg's Test** | **t-Egger's test** | ***P*-Egger's test** |
| --- | --- | --- | --- | --- | --- |
| allele T vs. allele C | 22 | 0.73 | 0.463 | 0.81 | 0.428 |
| carrier T vs. carrier C | 22 | 0.23 | 0.822 | 0.39 | 0.701 |
| TT vs. CC | 22 | 1.75 | 0.080 | 2.09 | 0.049 |
| TT vs. CC+CT | 22 | 1.64 | 0.102 | 2.14 | 0.044 |
| CT vs. CC | 22 | 0.90 | 0.367 | -0.39 | 0.698 |
| CT+TT vs. CC | 22 | 0.06 | 0.955 | 0.26 | 0.797 |

**Note:** N, number; z-Begg's Test, continuity corrected z value of Begg's Test; *P*-Begg's Test, continuity corrected

*P* value of Begg's Test; t-Egger's test, t value of Egger's Test; *P*- Egger's Test, *P* value of Egger's Test.
